# Association between gut microbiota and longevity: a genetic correlation and mendelian randomization study

**DOI:** 10.1186/s12866-022-02703-x

**Published:** 2022-12-13

**Authors:** Dan He, Li Liu, Zhen Zhang, Xuena Yang, Yumeng Jia, Yan Wen, Shiqiang Cheng, Peilin Meng, Chun’e Li, Huijie Zhang, Chuyu Pan, Feng Zhang

**Affiliations:** 1 Key Laboratory of Trace Elements and Endemic Diseases of National Health and Family Planning Commission, Xi’an Jiaotong University, 710061 Xi’an, China; 2 Key Laboratory of Environment and Genes Related to Diseases of Ministry of Education of China, Xi’an Jiaotong University, Xi’an, China

**Keywords:** Gut microbiota, Longevity, Linkage disequilibrium score regression, Mendelian randomization analysis, Aging-related disease

## Abstract

**Background:**

Longevity is one of the most complex phenotypes, and its genetic basis remains unclear. This study aimed to explore the genetic correlation and potential causal association between gut microbiota and longevity.

**Results:**

Linkage disequilibrium score (LDSC) regression analysis and a bi-directional two-sample Mendelian Randomization (MR) analysis were performed to analyze gut microbiota and longevity-related traits. LDSC analysis detected four candidate genetic correlations, including *Veillonella* (genetic correlation = 0.5578, *P* = 4.67 × 10^− 2^) and *Roseburia* (genetic correlation = 0.4491, *P* = 2.67 × 10^− 2^) for longevity, *Collinsella* (genetic correlation = 0.3144, *P* = 4.07 × 10^− 2^) for parental lifespan and *Sporobacter* (genetic correlation = 0.2092, *P* = 3.53 × 10^− 2^) for healthspan. Further MR analysis observed suggestive causation between *Collinsella* and parental longevity (father’s age at death) (weighted median: b = 1.79 × 10^− 3^, *P* = 3.52 × 10^− 2^). Reverse MR analysis also detected several causal effects of longevity-related traits on gut microbiota, such as longevity and *Sporobacter* (IVW: b = 7.02 × 10^− 1^, *P* = 4.21 × 10^− 25^). Statistical insignificance of the heterogeneity test and pleiotropy test supported the validity of the MR study.

**Conclusion:**

Our study found evidence that gut microbiota is causally associated with longevity, or vice versa, providing novel clues for understanding the roles of gut microbiota in aging development.

**Supplementary Information:**

The online version contains supplementary material available at 10.1186/s12866-022-02703-x.

## Introduction

Longevity is among the most complex phenotypes studied to date, for many complex factors affecting aging and life expectancy, including income, nutrition, education and health services [1]. Furthermore, a longer life does not necessarily mean a longer healthspan, with the most common complex diseases increasing with age. Previous studies have shown that genetic and environmental factors influence longevity and healthy aging. The heritability of age at death in adulthood is about 25% [2]. Among the non-genetic factors, social status, non-smoking, and diet behaviours significantly contribute to longevity [3].

As a complex and dynamic ecosystem, the gut microbiota is associated with major conditions like obesity, type 2 diabetes, cardiovascular disease and cancer [4–6]. Associations between aging and gut microbiota have been well-studied. Several studies have suggested that aging is associated with the composition of the gut microbiome and its metabolites, primarily through nutrient signaling pathways, immune regulation mechanisms, and epigenetic mechanisms [7–9]. Aging-related gut dysbiosis may lead to the occurrence or progression of other metabolic diseases, resulting in a loss of healthy longevity [10]. In addition, aging-related diet patterns can influence gut microbiota health, and dietary interventions can improve intestinal health and immune status in older adults, thereby increasing their healthy longevity [11]. However, the biological mechanism of gut microbiota affecting longevity-related traits, such as healthspan and longevity, remains elusive.

Genome-wide association study (GWAS) has been widely used for exploring candidate genetic variants for multiple complex traits and diseases, including longevity phenotypes and gut microbiota [12]. Genetic correlation is an important population parameter to describe the genetic correlation of different phenotypes. Its use may lead to a novel understanding of heredity and pathogenesis mechanisms for complex diseases [13]. Linkage disequilibrium score (LDSC) regression is a powerful tool to estimate genetic correlations across human complex traits based on GWAS summary data [14]. A major advantage of LDSC for large-scale analyses is the use of summary statistical data rather than individual-level genotype data, which makes data analysis more convenient, as most large GWAS analyses report summary statistical results. It has been widely used for identifying the shared genetic architecture of complex traits, including estimating the heritability of immune-related diseases and several psychiatric disorders and testing their genetic correlation [15].

As an epidemiological approach, mendelian randomization analyses (MR) enable an assessment of potential causation between exposures and outcomes based on observational data [16]. Compared to conventional epidemiologic studies, it can effectively avoid the influence of confounding factors and reverse causality on the study results. Suppose the genetic instruments used are associated with the exposure rather than with any confounders of the exposure-outcome relationship. In that case, the results from this MR analysis may provide strong evidence for causality. LDSC regression and MR analysis could investigate whether there is a common genetic background and potential causation between human phenotypes. For example, Adewuyi et al. identified shared loci between endometriosis and depression via LDSC regression and implicated association with gastric mucosa abnormalities in their causal pathways by performing MR analysis [17].

Using GWAS data from European ancestry, our study combined LDSC regression and two-sample MR analysis to explore the associations between gut microbiota and four longevity-related traits. We hope our analysis may provide novel clues for the effect of gut microbiota on the development of longevity.

## Methods

### Study design

**Fig. 1:**
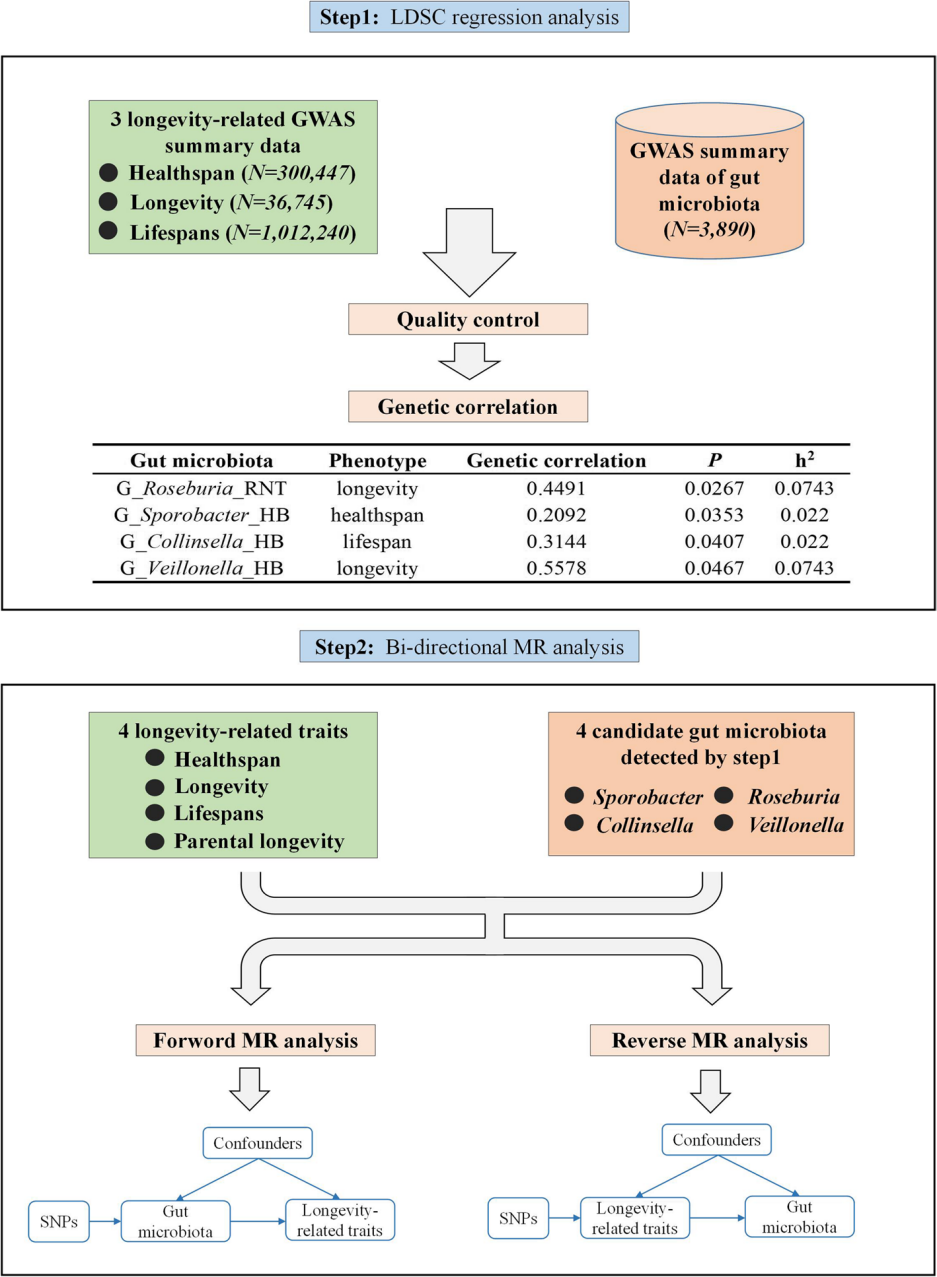
The overall design of this study

**Fig. 2:**
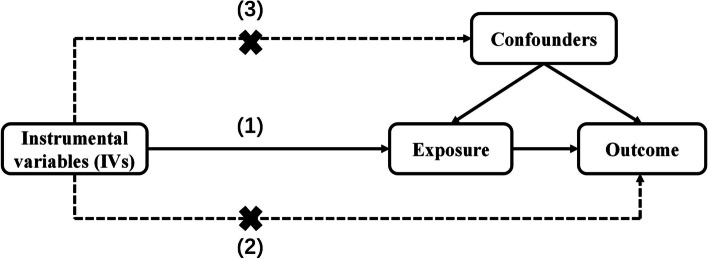
Basic principles of Mendelian randomization. Mendelian randomization can be used to test the hypothesis that gut microbiota causes longevity, provided that three key assumptions are met adequately: (1) the IVs are robustly associated with exposure; (2) the IVs are not associated with confounders; (3) the IVs have no association with the outcome except through the exposure

Figure 1 outlines this study’s overall design. Briefly, our study comprises two main steps: first, LDSC regression was conducted to explore the genetic correlation between three longevity-related traits and each gut microbiota; second, bi-directional MR analysis was performed to verify causal associations between candidate gut microbiota and longevity-related traits. Figure 2 shows the basic principles of MR analysis.

### GWAS data source

The GWAS data source of four longevity-related traits and gut microbiota was summarized in Table 1. All these GWAS data sources are summary statistics. LDSC analysis used GWAS data of healthspan, longevity, parental lifespan and gut microbiota, and MR analysis used GWAS data of four longevity-related traits and gut microbiota.

**Table 1:** The GWAS data source of four longevity-related traits and gut microbiota

Phenotype	GWAS data source	Sample size	Study or population	Database
Longevity	Deelen et al., 2019 [18]	36,745	Two meta-analyses in individuals of European ancestry	Longevity genomics website (https://www.longevitygenomics.org/downloads)
Healthspan	Zenin et al., 2019 [19]	300,447	UK Biobank	OpenAIRE (10.5281/zenodo.1302861)
Lifespan	Timmers et al., 2019 [20]	1,012,240	UK Biobank and 26 independent European-heritage population cohorts	Edinburgh DataShare (10.7488/ds/2463)
Parental longevity	Pilling et al., 2016 [21]	75,244	UK Biobank	MRC IEU OpenGWAS database (https://gwas.mrcieu.ac.uk/)
Gut microbiota	Hughes et al., 2020 [12]	3,890	The Flemish Gut Flora Project (FGFP)	University of Bristol data repository, data.bris, (10.5523/bris.22bqn399f9i432q56gt3wfhzlc)

#### GWAS data of healthspan, longevity, parental longevity and parental lifespans

Four publicly available GWAS datasets of longevity-related traits were used here, which are briefly described below:

The GWAS data of healthspan consists of 300,477 British-ancestry individuals from the UK Biobank (UKB) [19]. Healthspan is defined as the age at onset or death of the first disease (including cancer, stroke, dementia, diabetes, Congestive Heart Failure (CHF), Myocardial Infarction (MI), and Chronic Obstructive Pulmonary Disease (COPD). Genotype imputation was performed using IMPUTE2 and UK10K/1000 Genomes reference panel. The Cox-Gompertz survival model was used to predict the age corresponding to the end of the healthspan for each study individual. Covariates are age, sex, genotyping batch, assessment center, and 40 genetic principal components. Summary statistics retained 11,309,218 single nucleotide polymorphisms (SNPs). More details can be seen in the published article [19].

The GWAS data of parental lifespan consists of 1,012,240 European-ancestry individuals, including 512,047 mother and 500,193 father lifespans. Genotype imputation was carried out using IMPUTE4.0, with the reference panel of a combined UK10K/1000 Genomes. The cox survival model was used to perform a sex-stratified analysis for UKB individuals, adjusting for sex, ethnicity, assessment center, genotyping batch, and ten principal components. Then, combined the allelic effects in fathers and mothers into a single parental survival association in two ways. Thirdly, parent survival summary statistics from LifeGen (including 26 cohorts) were combined using the fixed-effects meta-analysis, adjusting for the correlation between survival traits. Summary statistics were available for 9,085,648 SNPs. Detailed descriptions of quality control, GWAS, and meta-analysis are available in the published article [20].

The GWAS data of parental longevity was collected for 75,244 British-ancestry individuals from the UKB. The analyses were performed separately on mother’s age at death (*N* = 52,776), father’s age at death (*N* = 63,775), and also on a combined phenotype (*N* = 45,627), excluding offspring of parents who died prematurely. Genotype imputation was performed using IMPUTE2 and UK10K/1000 Genomes reference panel. BOLT-LMM was used for GWAS to infer the association of variants with each phenotype, adjusting confounding factors (including sex, age, array type, assessment center, and the first five principal components). After quality control, 9,658,292 imputed SNPs were available for analysis. More details of the GWAS data can be found in the published article [21].

The GWAS data of longevity was derived from 36,745 individuals of European ancestry in multiple studies, including 11,262 cases and 25,483 controls [18]. Cases were individuals who lived to age above the 90th percentile, and the control group was the age of death (or age at last follow-up visit) at or before the 60th percentile age. For each cohort, genotype imputation was performed using the 1000 Genomes reference panel, and quality control of the summary statistics was performed using the EasyQC software. Logistic regression analysis was used to analyze GWAS for each cohort, adjusting for clinical site, known family relationships, and the first four principal components as covariates. A fixed effect meta-analysis combined GWAS of participating cohorts using METALs [22]. Summary statistics were available for 9,292,576 SNPs. Detailed descriptions of quality control, GWAS, and meta-analysis are available in the published article [18].

#### GWAS data of gut microbiota

The GWAS data on gut microbiota was obtained from the previous GWAS meta-analysis. [12]. Host genotype data was from 3,890 individuals of the Flemish Gut Flora Project (FGFP) (*N* = 2,223) and two German cohorts (including Food-Chain Plus (*N* = 950) and the PopGen cohort (*N* = 717)). FGFP genotype imputation was performed using IMPUTE2 and UK10K/1000 Genome Project phase 3 samples as the reference panel. The two German cohorts were genotyped using the Affymetrix Genome-Wide Human SNP Array 6.0 and the Illumina Omni Express + Exome array, respectively. Rank normal transformed (RNT) model and hurdle binary (HB) model were used to calculate the phenotype of microbial taxa. All microbiome abundance, α-diversity, and primary FGFP association analyses were performed using Snptest.2.5.0 [23], and the meta-analyses were performed in the software package META. After quality control, 7,711,310 SNPs were retained for the FGFP microbiome GWAS. More details on RNT and HB models can be found in the published article [12].

### LDSC regression analysis

LDSC regression analysis is a new method for estimating genetic correlation, which requires only GWAS summary statistics. Due to the limited GWAS summary data for parental longevity, LDSC analysis in this study was only conducted to assess the genetic correlation of gut microbiota with healthspan, longevity and lifespan. Firstly, munge_sumstats.py (https://github.com/bulik/ldsc/blob/master/munge_sumstats.py) was used to reformat summary statistics and remove variants that are not SNPs (e.g., indels), strand ambiguous SNPs, and repeated SNPs. SNPs with imputation quality score > 0.9 and MAF > 0.01 were selected in our study to prevent bias from variable imputation quality. After quality control, 1,226,476 SNPs for longevity, 1,182,992 SNPs for healthspan, 1,167,117 SNPs for lifespan, and about 1.2 million SNPs for each gut microbiota were retained. Secondly, following the standard approach recommended by the developers, the 1000 Genomes Project was used as the linkage disequilibrium (LD) reference panel for estimating the LD score. Thirdly, we examined the genetic correlation between gut microbiota and longevity-related traits using LDSC (https://github.com/bulik/ldsc) (LD SCore v1.0.1). 157 gut microbiota and three longevity-related traits were applied in our LDSC analyses, and a strict Bonferroni threshold was set to *P* < 1.06 × 10^− 4^ (0.05/471). However, after the Bonferroni correction, there was no significant correlation. Therefore, we set a candidate threshold of LDSC regression analysis at *P* < 0.05 and used MR analysis to verify the causal association between candidate gut microbiota and longevity-related traits.

### Genetic instruments selection

MR analysis used genetic variants associated with exposure as instrumental variables (IVs). In this study, candidate gut microbiota associated with longevity were detected by LDSC regression analysis, and then SNPs of those candidate gut microbiota were used as genetic instruments. Appropriate SNPs used as IVs must be robustly associated with gut microbiota, so we selected independent SNPs related to gut microbiota at the genome-wide significant level (*P* < 5 × 10^–8^). Fifty-eight independent SNPs were extracted from gut microbiota GWAS studies for SNP exposure in this analysis, including 24 SNPs for *Collinsella*, 33 SNPs for *Sporobacter* and 1 SNP for *Veillonella* (Table S1).

### Mendelian randomization (MR) analysis

MR analysis was performed to assess the causation between candidate gut microbiota and longevity-related traits. The inverse variance weighting (IVW) method was used to analyze gut microbiota’s causal effect on longevity-related traits. To test the validity of our findings, the sensitivity analyses of the weighted median, weighted mode and MR-Egger regression for the causal effect estimation of gut microbiota on longevity-related traits were also performed here [24]. Egger regression was used to account for uncorrelated pleiotropy, and weighted median, and the weighted mode were used to account for correlated pleiotropy. In the case of exposure with only one significant SNP in the GWAS, the Wald ratio model was used for MR analysis. In addition, to investigate whether longevity would affect the gut microbiota, a reverse MR analysis was also performed in the same way. All statistical analyses were conducted in Two Sample MR package of R software [25].

## Results

### Genetic correlation between gut microbiota and longevity

**Fig. 3:**
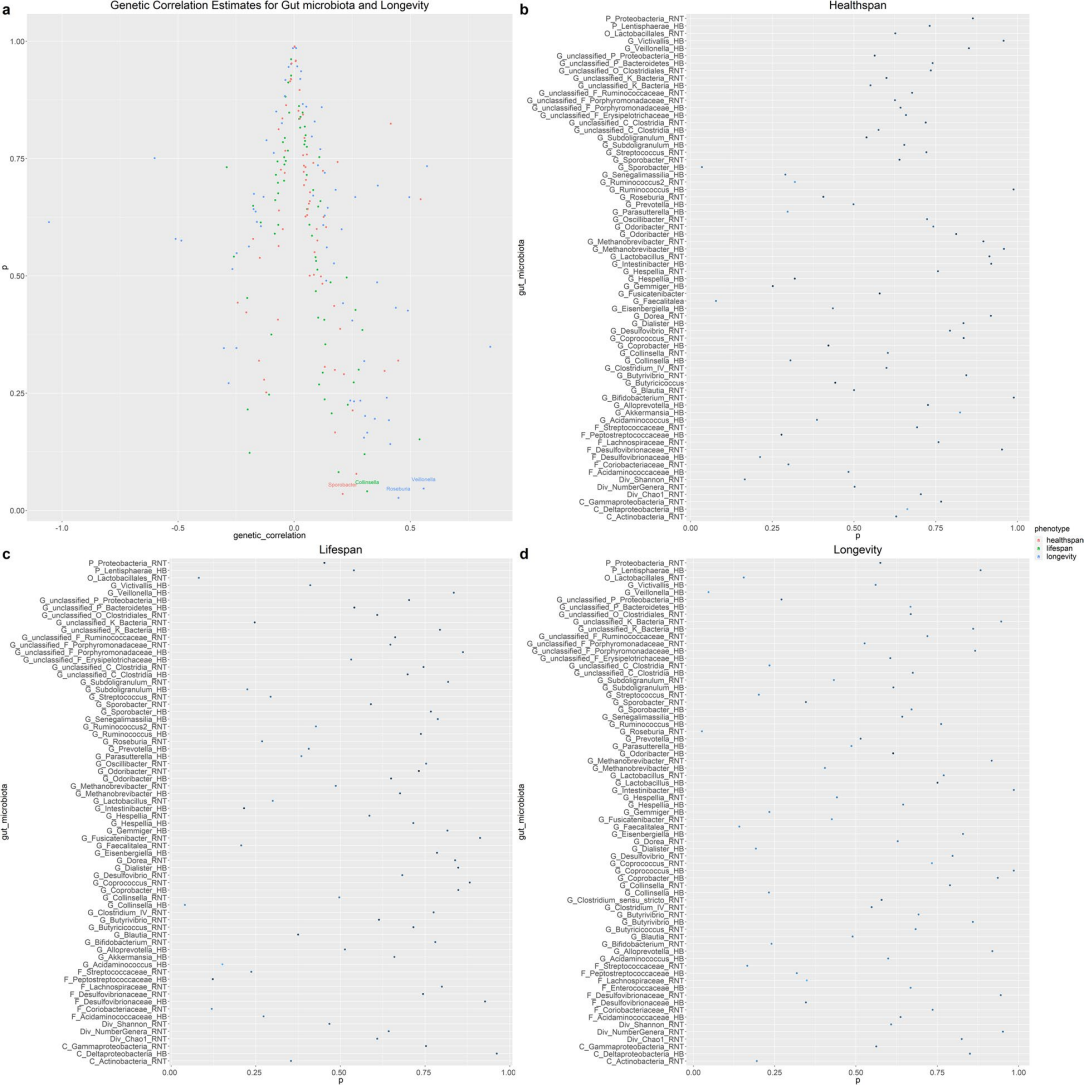
Genetic correlation estimates for gut microbiota and longevity by LDSC regression analysis. **a **Circle color indicates the phenotype of longevity, whilethe red plot represents the healthspan, the green plot represents the parental lifespan,and the blue plot represents the parental longevity.Geneticcorrelation estimates with *P*<0.05 were annotated in the figure. All the genetic correlationsfor gut microbiota and longevity illustrated here can be found in Supplementary Table 2. **b**, **c**, **d** Circles represent the genetic correlation of healthspan, lifespan and longevity, respectively. The circle color indicates the degree of genetic correlation. The dark color has a high degree of genetic correlation, while the light color has a low degree of genetic correlation

LDSC detected 4 candidate genetic correlation between gut microbiota and longevity-related traits (Table 2), such as *Veillonella* (genetic correlation = 0.5578, *P* = 4.67 × 10^− 2^) and *Roseburia* (genetic correlation = 0.4491, *P* = 2.67 × 10^− 2^) for longevity, *Collinsella* (genetic correlation = 0.3144, *P* = 4.07 × 10^− 2^) for parental lifespan, and *Sporobacter* (genetic correlation = 0.2092, *P* = 3.53 × 10^− 2^) for healthspan. Figure 3 and Table S2 show the results of LDSC regression analysis.

**Table 2:** Four candidate genetic correlations between gut microbiota and longevity-related traits

Gut microbiota	Phenotype	Genetic correlation	*P*
*G_Roseburia_RNT*	Longevity	0.4491	2.67 × 10^− 2^
*G_Sporobacter_HB*	Healthspan	0.2092	3.53 × 10^− 2^
*G_Collinsella_HB*	Lifespan	0.3144	4.07 × 10^− 2^
*G_Veillonella_HB*	Longevity	0.5578	4.67 × 10^− 2^

*G* Genus, *RNT* Rank-normal transformation, *HB* Hurdle binary

### Influence of gut microbiota on longevity-related traits

**Fig. 4:**
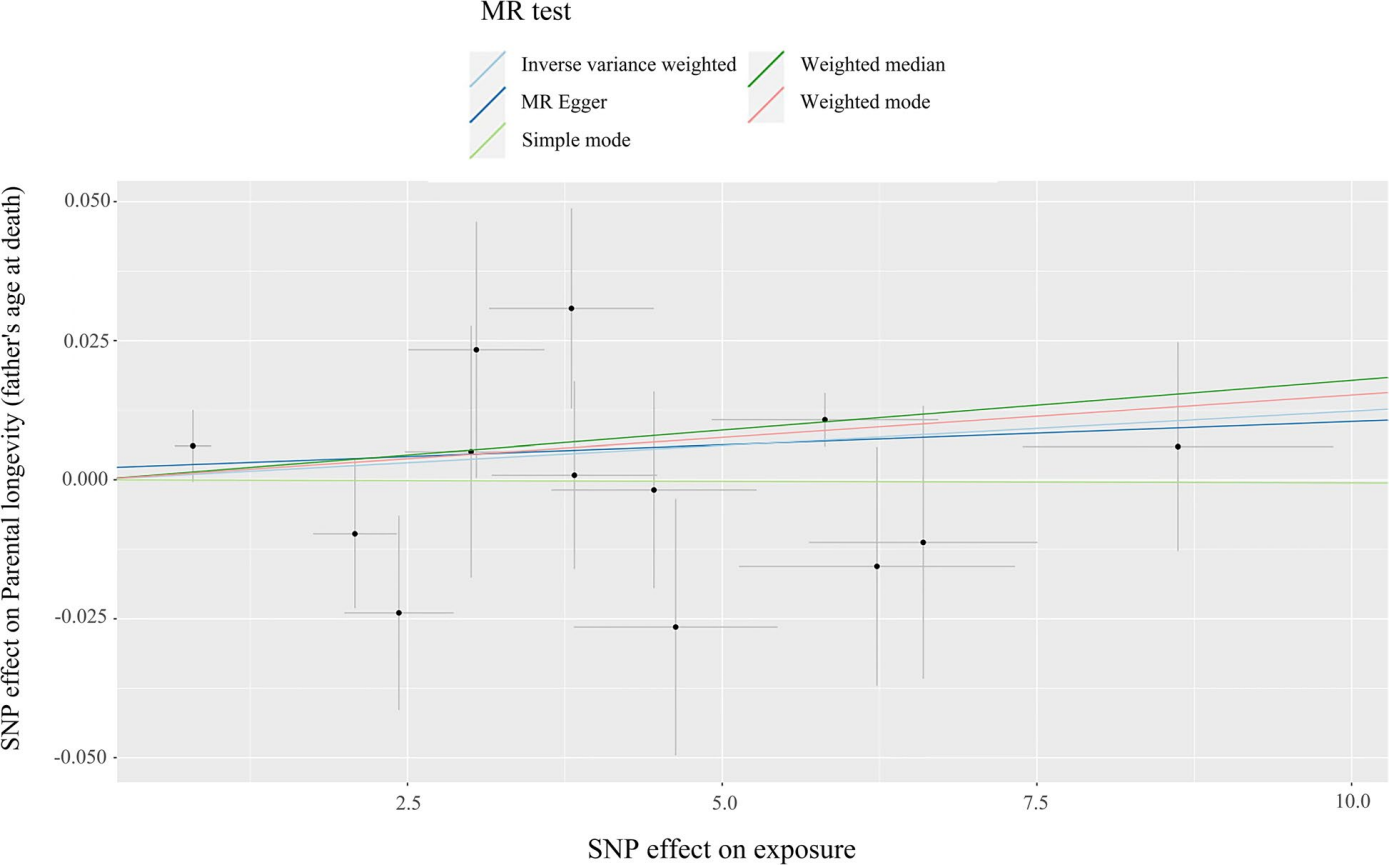
Scatter plots of the association of *Collinsella *and parental longevity. Each black point represents an SNP, plotted by the estimate of SNP on *Collinsella* (x-axis) and the estimate of SNP on parental longevity (father’s age at death) (y-axis). The slopes of each line represent the potential causal associations for each method

For MR analysis of gut microbiota on longevity-related traits, a suggestive causal association was found in the weighted median method (Table 3; Fig. 4), showing a positive causal association between *Collinsella* and parental longevity (father’s age at death) (b = 0.0018, *P* = 3.52 × 10^− 2^). In addition, the *P*-value of the intercept term in MR-Egger (*P* = 7.57 × 10^− 1^) supported that our MR study was not affected by horizontal pleiotropy. The heterogeneity test highlighted that there is no existence of heterogeneity (MR-Egger: *P* = 4.03 × 10^− 1^, IVW: *P* = 5.07 × 10^− 1^). Moreover, *Veillonella* also was associated with lifespan(b=-0.0243, *P* = 1.52 × 10^− 2^), healthspan(b=-0.0259, *P* = 2.67 × 10^− 2^), mother’s attained age (b = 0.0073, *P* = 1.43 × 10^− 2^) and parental longevity (b = 0.0064, *P* = 3.62 × 10^− 2^) using Wald ratio (IVW method was not applicable). All results of MR analysis were summarized in Table S3.

**Table 3:** Causal effects of gut microbiota on longevity-related traits estimated by MR analysis

**MR**							**Heterogeneity**	**Pleiotropy**
**Exposure**	**Outcome**	**Method**	**N**_**SNP**_	**b**	**Se**	***P***	***P***	***P***
*G_Collinsella_HB*	Parental longevity (father's age at death)	MR Egger	13	8.38×10^-4^	1.42×10^-3^	5.66×10^-1^	4.30×10^-1^	7.57×10^-1^
		Weighted median	13	1.79×10^-3^	8.48×10^-4^	3.52×10^-2^		
		Inverse variance weighted	13	1.23×10^-3^	6.86×10^-4^	7.29×10^-2^	5.07×10^-1^	
		Simple mode	13	-5.42×10^-5^	2.06×10^-3^	9.79×10^-1^		
		Weighted mode	13	1.52×10^-3^	7.95×10^-4^	8.00×10^-2^		
*G_Veillonella_HB*	Parental longevity (mother's attained age)	Wald ratio	1	7.27×10^-3^	2.96×10^-3^	1.43×10^-2^		
	Lifespan	Wald ratio	1	-2.43×10^-2^	1.00×10^-2^	1.52×10^-2^		
	Healthspan	Wald ratio	1	-2.59×10^-2^	1.17×10^-2^	2.67×10^-2^		
	Parental longevity (combined parental attained age, Martingale residuals)	Wald ratio	1	6.36×10^-3^	3.03×10^-3^	3.62×10^-2^		

*G* Genus, *RNT* Rank-normal transformation, *HB* Hurdle binary; b means the estimated causal effect

### Influence of longevity-related traits on gut microbiota

Several reverse causal effects of longevity-related traits on gut microbiota were observed in our reverse MR analyses (Table 4). For example, longevity (IVW: b=-0.3096, *P* = 1.43 × 10^− 5^) and healthspan (Weighted mode: b=-1.4024, *P* = 4.40 × 10^− 2^) were found to have causal associations with *Collinsella.* In addition, significant associations were also found in longevity-related traits on *Sporobacter*, such as longevity associated with *Sporobacter* were observed in all our MR methods (*P* < 6.28 × 10^− 4^). All tests of pleiotropy and heterogeneity were negative, indicating that our MR results were not biased by heterogeneity or horizontal pleiotropy. The results of all reverse MR analyses were summarized in Table S4.

**Table 4:** Causal effects of longevity-related traits on gut microbiota estimated by MR analysis

**MR**							**Heterogeneity**	**Pleiotropy**
**Exposure**	**Outcome**	**Method**	**N**_**SNP**_	**b**	**Se**	***P***	***P***	***P***
Longevity	*G_Collinsella_HB*	MR Egger	42	-0.20	0.16	2.05×10^-1^	9.99×10^-1^	4.60×10^-1^
		Weighted median	42	-0.31	0.09	1.05×10^-3^		
		Inverse variance weighted	42	-0.31	0.07	1.43×10^-5^	9.99×10^-1^	
		Simple mode	42	-0.31	0.16	5.42×10^-2^		
		Weighted mode	42	-0.28	0.14	4.41×10^-2^		
Healthspan	*G_Collinsella_HB*	MR Egger	331	-1.19	0.74	1.07×10^-1^	1.00	1.08×10^-1^
		Weighted median	331	-0.09	0.24	7.18×10^-1^		
		Inverse variance weighted	331	-0.04	0.18	8.39×10^-1^	1.00	
		Simple mode	331	1.31	0.76	8.61×10^-2^		
		Weighted mode	331	-1.40	0.69	4.40×10^-2^		
Longevity	*G_Sporobacter_HB*	MR Egger	42	0.84	0.15	2.05×10^-6^	9.85×10^-1^	3.22×10^-1^
		Weighted median	42	0.66	0.10	7.24×10^-12^		
		Inverse variance weighted	42	0.70	0.07	4.21×10^-25^	9.84×10^-1^	
		Simple mode	42	0.61	0.16	6.28×10^-4^		
		Weighted mode	42	0.68	0.14	1.79×10^-5^		
Parental longevity (combined parental age at death)	*G_Sporobacter_HB*	MR Egger	6	15.08	5.08	4.13×10^-2^	5.39×10^-1^	5.93×10^-2^
		Weighted median	6	1.68	2.84	5.55×10^-1^		
		Inverse variance weighted	6	2.63	2.50	2.92×10^-1^	7.72×10^-2^	
		Simple mode	6	-1.00	5.39	8.61×10^-1^		
		Weighted mode	6	-1.78	5.10	7.41×10^-1^		
Parental longevity (combined parental attained age, Martingale residuals)	*G_Sporobacter_HB*	MR Egger	10	-7.63	3.11	3.96×10^-2^	8.54×10^-1^	6.22×10^-2^
		Weighted median	10	-1.00	2.18	6.46×10^-1^		
		Inverse variance weighted	10	-1.78	1.54	2.47×10^-1^	4.63×10^-1^	
		Simple mode	10	0.98	4.24	8.22×10^-1^		
		Weighted mode	10	-6.41	2.49	3.01×10^-2^		
Parental longevity (both parents in top 10%)	*G_Sporobacter_HB*	MR Egger	6	14.23	5.22	5.26×10^-2^	7.47×10^-1^	9.08×10^-2^
		Weighted median	6	5.05	3.35	1.32×10^-1^		
		Inverse variance weighted	6	4.17	3.02	1.68×10^-1^	2.31×10^-1^	
		Simple mode	6	2.04	7.42	7.94×10^-1^		
		Weighted mode	6	8.54	3.30	4.91×10^-2^		
Parental longevity (95 years and older)	*G_Sporobacter_HB*	Wald ratio	1	51.75	25.07	3.90×10^-2^		
Parental longevity (mother's age at death)	*G_Sporobacter_HB*	Wald ratio	1	11.00	4.04	6.41×10^-3^		

*G* Genus, *RNT* Rank-normal transformation, *HB* Hurdle binary; b means the estimated causal effect

## Discussion

The aging population has led to a higher prevalence of chronic diseases in recent years, and the increased burden on health care systems in developing countries has made it necessary to explore how to extend a healthy lifespan [26]. By conducting a multivariate meta-analysis of three European-ancestry GWAS with aging traits: healthspan, parental lifespan and longevity, Timmers et al. found genetic correlations among the three, with longevity most closely related to parental lifespan. In addition, their analysis further identified 78 genes associated with these three phenotypes as well as various aging pathways [27]. Furthermore, the gut microbiota has been implicated in aging, providing potential targets for novel interventions to promote healthy aging [28]. Our study explored the effect of gut microbiota on longevity based on the data from multiple independent large-scale GWAS of gut microbiota and longevity. Findings from the LDSC regression analysis indicate a suggestive genetic correlation between gut microbiota and longevity. Utilizing the independent GWAS data, we further tested the causal association between identified candidate gut microbiota and longevity-related traits using MR analysis. Our results provided potential clues for the genetic pathogenies of the effect of gut microbiota on longevity.

Previous studies found that community richness measures and enriched beneficial bacteria could be seen as microbial signatures of longevity, providing a promising target for promoting healthy aging [29]. In an experimental study of faecal microbial transplantation (FMT), Bárcena et al. found intestinal dysbiosis existed in both progeroid mouse models, and FMT from wild-type mice enhanced both healthspan and lifespan. Consistent with the mouse models, human progeria patients also showed intestinal dysbiosis, with a substantial increase in Verrucomicrobia and a reduction in Proteobacteria in human longevity [30]. Gut microbiota may influence the healthy lifespan of the elderly through the following biological mechanisms: (1) gut microbiota can modulate the immune response, primarily by maintaining a balance between the inflammatory and anti-inflammatory networks [7, 31]; (2) the role of the microbiota-gut-brain axis, including intestinal dysbiosis influences on metabolic diseases, such as digestive diseases and mental illness [10, 32, 33]; (3) genetic and epigenetic regulation of aging, including through oxidative stress induced cellular senescence [34]; (4) nutrition is a common factor linking the gut microbiota to the host genome, such as extend lifespan through caloric restriction [9, 35].

We identified several candidate longevity-related gut microbiota and observed suggestive causation between *Collinsella* and parental longevity. The role of gut microbiota in various chronic metabolic diseases and aging in longevity research occupies an important position. As metabolic diseases and aging are both characterized by low-grade inflammation and activation of the innate immune system, the influence of gut microbiota on obesity and related metabolic disorders may help detect healthy aging [36]. A study of gut microbiome patterns revealed a positive association between microbial metabolic markers and gut microbiome uniqueness, reflecting a healthy aging phenotype and predicting longevity in older adults [37]. Centenarians have a lower incidence of chronic illness and an extended healthspan [38]. By producing ursodeoxycholate, *Collinsella* inhibits the expression of pro-inflammatory cytokines and has antioxidant and anti-apoptotic effects [39]. The abundance of *Collinsella* was significantly higher in South Korean centenarians than in normal elderly individuals based on a century-healthy aging model [40]. Notably, although *Collinsella* has recently been associated with metabolic regulation, atherosclerosis and type 2 diabetes mellitus, dietary interventions strongly influence *Collinsella*, and further research is needed to explore its pathogenic mechanism [41]. Scientific evidence has shown that the Mediterranean diet can affect human life expectancy by preventing the development of chronic metabolic diseases and reducing cancer incidence [42]. Our results hope to provide biomarkers for clinicians and researchers to assess aging status by detecting changes in gut microbiota.

The *Sporobacter*, belonging to the family *Ruminococcaceae*, can degrade aromatic compounds and produce short-chain fatty acids (SCFAs) [43]. SCFAs as the end-products of fermentation of dietary fibers and resistant starch, which play an important role in appetite regulation, energy metabolism, inflammation and disease [44–46]. For example, in a study of gut microbiota in children with irritable bowel syndrome (IBS), researchers gave a low fermentable substrate diet (LFSD) to IBS children. They identified significantly increased abundance of *Sporobacter* in children who responded to the diet [47]. A healthy immune system protects the body, and aging results from a decline in immune function, controlled by the thymus, central nervous system, and pineal gland. A number of immune-mediated inflammatory diseases are associated with gut microbiota from an immunological perspective, including ulcerative colitis (UC), multiple sclerosis (MS), rheumatoid arthritis (RA), and Crohn’s disease (CD), with a significant reduction in *Sporobacter* abundance in these conditions [48]. Diet supplementation of resin acid-enriched composition increased sow colostrum immunoglobulin G content and changed sow gut microbiota abundance, including a significant decrease in the abundance of *Sporobacter* [49]. In addition, *Sporobacter* is considered a critical bacteria for controlling intestinal infection or inflammation in treating non-infectious colitis with FMT [50]. Lin et al. found that the richness and diversity of *Sporobacter* increased significantly in patients with early gastric cancer after subtotal gastrectomy [51]. From what has been discussed above, we speculated that the genetic association between *Sporobacter* and longevity might be due to their shared influence on immune function and immune-mediated inflammatory diseases.

*Veillonella* was also observed to have causal effects on longevity in our study, with a higher correlation for longevity. There was also an increased abundance of *Veillonella* in patients with early gastric cancer after subtotal gastrectomy due to the decreased stomach volume and increased stomach pH after subtotal gastrectomy [51]. It is well known that *Veillonella* is a pro-inflammatory bacterium causing severe acute and chronic infections, such as chronic anaerobic pneumonitis [52] and osteomyelitis [53]. Gut microbiota may be a pathogenic factor of colorectal cancer (CRC), and *Veillonella* has been found to contribute to CRC in humans [54]. Dayama et al. found that *Veillonella* is positively related to DUOXA2 in cystic fibrosis (CF) patients, through DUOXA2 in ulcerative colitis organization specificity to participate in the inflammatory response, thus leading to colorectal cancer, a complication of CF [55]. Furthermore, *Pseudomonas* has demonstrated an increased virulence in the presence of *Veillonella*, resulting in a deterioration of clinical condition [56]. Tumor-suppressor genes can be classified as longevity assurance genes, and cancer was considered a natural ceiling on human longevity and its incidence rose exponentially with age [57]. Our findings emphasized the importance of the interaction of the gut microbiota with host genes for cancer and human longevity. Notably, since we only performed the Wald Ratio model for MR analysis, even though the detection of causal associations between *Veillonella* and three longevity-related traits can greatly support the reliability of our analysis, further studies still need to examine whether *Veillonella* is causally associated with longevity carefully.

Since *Roseburia* did not have relevant SNPs as IVs, we could not test the causal association between *Roseburia* and longevity-related traits. However, LDSC regression analysis found that apart from the other three gut microbiota associated with longevity, there was also a strong genetic correlation between *Roseburia* and longevity. As a common beneficial flora, *Roseburia spp.* produces SCFAs, especially butyrate, which promote gut ecosystems, immunity, and anti-inflammatory activity, leading to an improvement in chronic conditions such as atherosclerosis and alcoholic fatty liver [58]. Imhann et al. emphasized the importance of considering the interaction of gut microbiota and host genes as they relate to immune system function, with a higher number inflammatory bowel disease (IBD) genetic risk variants associated with a decrease in the abundance of *Roseburia* [59]. In addition, Wang et al. revealed a higher abundance of *Roseburia* and *Escherichia* in Chinese centenarians [60].

As our research showed, gut microbiota variability may potentially affect human health. However, due to the little known about gut microbiota, there is still a lot of scope for research on the association between gut microbiota and longevity. With the increase of age, longevity would reversely influence gut microbiota, which mainly includes two aspects. On the one hand, the abundance of antibiotic resistance genes (ARG) is a cumulative effect related to age, and antibiotic resistance in bacteria is a major factor influencing gut microbiota composition [61]. On the other hand, the longevity process has been shown to have a profound effect on the composition and structure of gut microbiota by regulating host metabolisms, such as lactobacillus intake and defecation frequency [62]. Indeed, our reverse MR analysis results found several causal effects of longevity-related traits on gut microbiota, suggesting that there may be a bi-directional causal association between gut microbiota and longevity. However, the clear association between gut microbiota and longevity is poorly understood, and we need to collect novel, larger samples and conduct experiments to investigate whether longevity may reverse the effects of gut microbiota on longevity in our future studies.

Our genetic analysis of gut microbiota and longevity was based on large-scale GWAS data sources. It can largely mitigate confounding factors such as environment and lifestyles, making the results relatively reliable. In addition, we performed LDSC regression analysis and MR analysis in different longevity cohorts, which makes genetic analysis more convincing. However, there are also several limitations in this study. Firstly, multiple test correction is necessary. The purpose of LDSC analysis in this study was to preliminarily screen the candidate gut microbiota related to longevity and verify it by MR analysis. As the LDSC results after the Bonferroni correction were insignificant, we need to search for more biological evidence from larger samples to validate our findings. Secondly, the available GWAS data from GWAS Catalog contained a small number of SNPs for parental longevity, leaving the estimated heritability to be out of bounds for genetic correlation analysis. Therefore, we could only assess the genetic association of gut microbiota with healthspan, longevity and lifespan. Thirdly, although our study fully used the largest publicly available GWAS giving adequate power, weak instruments would exaggerate the association between gut microbiota and longevity. In addition, the GWAS of *Veillonella* did not contain enough significant SNPs to conduct MR analysis, highlighting the need to include gut microbiota and longevity in larger-scale GWAS studies to explore their causal association thoroughly. Fourthly, the accuracy of our LDSC regression analyses might be affected by the power of the GWASs we used. To ensure statistical efficiency, we reformatted summary statistics, and inappropriate SNPs were filtered out of the analysis. However, considering the heterogeneity of GWAS data we used, further large-scale population studies incorporating various longevity phenotypes are warranted. Finally, longevity is regulated by genetic and environmental factors, and gut microbiota only plays a partial role and is greatly influenced by dietary behaviors.

## Conclusion

In conclusion, we tested genetic correlation and causal association between gut microbiota and longevity using LDSC regression and MR analyses of large GWAS data. Our study supports the potential role of gut microbiota in the development of longevity. Notably, further research is needed on the biological mechanisms by which gut microbiota influences longevity.

## Supplementary Information

**Additional file 1: Supplementary Table 1.** SNPs selected for instrumental variables.

**Additional file 2: Supplementary Table 2.** Genetic Correlation Estimates for Gut microbiota and Longevity by LDSC regression analysis.

**Additional file 3: Supplementary Table 3.** The MR analysis results of gut microbiota on longevity-related traits.

**Additional file 4: Supplementary Table 4.** The reverse MR analysis results of longevity-related traits on gut microbiota.

## Data Availability

The healthspan, lifespan, longevity and parental longevity GWAS summary statistics are available from OpenAIRE (10.5281/zenodo.1302861), Edinburgh DataShare (10.7488/ds/2463), the longevity genomics website (https://www.longevitygenomics.org/downloads) and MRC IEU OpenGWAS database (https://gwas.mrcieu.ac.uk/), respectively. All microbiome GWAS summary statistics are available online at the University of Bristol data repository with the identifier 10.5523/bris.22bqn399f9i432q56gt3wfhzlc.
