# Supplementary material for: Association between gut microbiota and longevity: a genetic correlation and mendelian randomization study
Source: BMC Microbiol. 2022 Dec 13;22:302. doi: 10.1186/s12866-022-02703-x (PMC9746102; doi:10.1186/s12866-022-02703-x)
Supplement: Supplementary file 1 — Additional file 1: Supplementary Table 1. SNPs selected for instrumental variables. [file 12866_2022_2703_MOESM1_ESM.docx]

**Supplementary table 1.** SNPs selected for instrumental variables.

| **Gut microbiota** | **SNP** | **Chr** | **FRQ** | **OR** | ***P*** | **Beta** | **Se** |
| --- | --- | --- | --- | --- | --- | --- | --- |
| *Collinsella* | rs149111954 | 2 | 0.0151 | 0.3837 | 6.45E-09 | -3.8018 | 0.655 |
|  | 3:33251775 | 3 | 0.0114 | 0.3347 | 1.19E-08 | -3.8046 | 0.6673 |
|  | 3:109808463 | 3 | 0.0482 | 0.5797 | 2.27E-08 | -2.3351 | 0.4177 |
|  | rs182546125 | 3 | 0.0103 | 0.2562 | 1.08E-09 | -3.9746 | 0.652 |
|  | rs117750622 | 4 | 0.0136 | 0.4414 | 4.53E-08 | -4.4574 | 0.815 |
|  | rs142747606 | 4 | 0.0135 | 0.378 | 3.18E-08 | -3.769 | 0.6814 |
|  | rs116310687 | 5 | 0.0137 | 0.4756 | 2.80E-12 | -8.6204 | 1.2337 |
|  | rs12515940 | 5 | 0.0125 | 0.2888 | 1.81E-08 | -3.0467 | 0.5412 |
|  | rs58434653 | 7 | 0.1488 | 0.5586 | 4.01E-08 | -0.7949 | 0.1448 |
|  | rs117045106 | 7 | 0.0253 | 0.3079 | 3.85E-10 | -2.0817 | 0.3325 |
|  | rs183064099 | 9 | 0.0109 | 0.2694 | 5.14E-09 | -3.422 | 0.5857 |
|  | 10:76397434 | 10 | 0.015 | 0.4413 | 3.25E-08 | -4.4669 | 0.8081 |
|  | rs373695493 | 11 | 0.01 | 0.2997 | 3.47E-09 | -4.2216 | 0.7146 |
|  | 11:80253922 | 11 | 0.0188 | 0.5961 | 6.24E-10 | -6.6582 | 1.0766 |
|  | rs145846445 | 11 | 0.0119 | 0.2587 | 1.14E-08 | -3.0048 | 0.5264 |
|  | rs186489504 | 12 | 0.013 | 0.2878 | 1.07E-12 | -4.5449 | 0.6382 |
|  | rs137896225 | 14 | 0.0132 | 0.502 | 1.33E-08 | -6.2288 | 1.0962 |
|  | rs11633283 | 15 | 0.0196 | 0.3264 | 2.15E-08 | -2.4311 | 0.4342 |
|  | rs79680858 | 15 | 0.0196 | 0.3265 | 1.99E-08 | -2.4503 | 0.4366 |
|  | rs192516752 | 15 | 0.0122 | 0.3509 | 3.69E-13 | -6.5966 | 0.9078 |
|  | rs144463940 | 16 | 0.0104 | 0.3484 | 1.07E-08 | -4.6299 | 0.8095 |
|  | 16:53102904 | 16 | 0.0161 | 0.4693 | 2.94E-08 | -4.1404 | 0.7467 |
|  | rs112012544 | 17 | 0.0123 | 0.3666 | 1.02E-10 | -5.8156 | 0.8997 |
|  | rs71364181 | 17 | 0.0142 | 0.3695 | 5.88E-09 | -3.8256 | 0.6573 |
| *Sporobacter* | 1:24608708 | 1 | 0.0102 | 0.4754 | 1.79E-08 | -6.7839 | 1.2048 |
|  | rs191328591 | 1 | 0.0112 | 0.4788 | 2.24E-09 | -7.1275 | 1.192 |
|  | rs181907827 | 1 | 0.011 | 0.4881 | 2.30E-08 | -6.4896 | 1.1614 |
|  | rs139604759 | 1 | 0.0107 | 0.2634 | 8.85E-13 | -5.0216 | 0.7026 |
|  | rs72770223 | 1 | 0.012 | 0.3818 | 7.98E-09 | -4.217 | 0.731 |
|  | rs75801886 | 2 | 0.0107 | 0.3781 | 1.10E-10 | -6.0307 | 0.9346 |
|  | rs115969665 | 2 | 0.0186 | 0.3505 | 2.17E-08 | -2.549 | 0.4554 |
|  | 2:103067545 | 2 | 0.0152 | 0.4314 | 3.33E-08 | -3.8742 | 0.7014 |
|  | rs115307907 | 2 | 0.0169 | 0.3345 | 5.56E-09 | -2.8273 | 0.485 |
|  | rs115149137 | 5 | 0.0128 | 0.4419 | 1.49E-09 | -5.1713 | 0.8554 |
|  | rs79093667 | 5 | 0.011 | 0.4719 | 6.84E-12 | -8.9499 | 1.3045 |
|  | rs13437089 | 6 | 0.0122 | 0.4631 | 4.14E-08 | -5.0486 | 0.9204 |
|  | rs10263895 | 7 | 0.0252 | 0.4684 | 2.59E-08 | -2.6174 | 0.4702 |
|  | rs76983443 | 8 | 0.012 | 0.3501 | 3.13E-09 | -4.261 | 0.7192 |
|  | rs117055488 | 9 | 0.0105 | 0.2843 | 2.13E-08 | -3.2781 | 0.5853 |
|  | rs147482387 | 9 | 0.011 | 0.3482 | 8.53E-09 | -3.925 | 0.6817 |
|  | rs41307449 | 9 | 0.0108 | 0.349 | 2.48E-08 | -3.7386 | 0.6706 |
|  | rs190460661 | 9 | 0.0189 | 0.4484 | 3.23E-09 | -3.7813 | 0.6388 |
|  | rs137875211 | 9 | 0.0111 | 0.3073 | 1.71E-09 | -3.7973 | 0.6305 |
|  | rs200993981 | 11 | 0.0102 | 0.4049 | 1.56E-10 | -6.682 | 1.0442 |
|  | rs143040115 | 11 | 0.0156 | 0.448 | 1.74E-08 | -4.4613 | 0.7915 |
|  | rs182301480 | 11 | 0.0153 | 0.4473 | 2.77E-11 | -6.2139 | 0.9332 |
|  | rs147105456 | 12 | 0.0103 | 0.3404 | 6.00E-09 | -4.3031 | 0.7398 |
|  | rs146462455 | 12 | 0.0102 | 0.3368 | 5.05E-09 | -4.3501 | 0.7442 |
|  | 14:37831424 | 14 | 0.0181 | 0.4542 | 1.84E-08 | -3.8066 | 0.6766 |
|  | rs147362343 | 17 | 0.013 | 0.333 | 4.40E-09 | -3.4643 | 0.5903 |
|  | rs34242502 | 17 | 0.0205 | 0.5079 | 5.56E-10 | -4.3422 | 0.7001 |
|  | 17:17903342 | 17 | 0.0326 | 0.5797 | 4.53E-08 | -2.7029 | 0.4942 |
|  | rs148318670 | 17 | 0.011 | 0.3105 | 1.17E-08 | -3.6537 | 0.6406 |
|  | rs62064461 | 17 | 0.0172 | 0.4547 | 1.59E-11 | -5.3776 | 0.798 |
|  | rs143838533 | 20 | 0.0101 | 0.408 | 9.15E-16 | -11.5574 | 1.4379 |
|  | rs117184659 | 20 | 0.0126 | 0.2926 | 1.32E-11 | -4.3026 | 0.6359 |
|  | rs140225883 | 21 | 0.0148 | 0.4357 | 6.44E-09 | -4.2738 | 0.7362 |
| *Veillonella* | rs117338748 | 15 | 0.019 | 2.7405 | 4.00E-08 | 1.5453 | 0.2814 |
