# Supplementary material for: Association between gut microbiota and longevity: a genetic correlation and mendelian randomization study
Source: BMC Microbiol. 2022 Dec 13;22:302. doi: 10.1186/s12866-022-02703-x (PMC9746102; doi:10.1186/s12866-022-02703-x)
Supplement: Supplementary file 2 — Additional file 2: Supplementary Table 2. Genetic Correlation Estimates for Gut microbiota and Longevity by LDSC regression analysis. [file 12866_2022_2703_MOESM2_ESM.docx]

**Supplementary table 2**. Genetic Correlation Estimates for Gut microbiota and Longevity by LDSC regression analysis

| **Gut_microbiota** | **Phenotype** | | **Genetic correlation** | ***P*** | **h2** |
| --- | --- | --- | --- | --- | --- |
| *G_Roseburia_RNT* | Longevity | 0.4491 | | 0.0267 | 0.0743 |
| *G_Sporobacter_HB* | Healthspan | 0.2092 | | 0.0353 | 0.022 |
| *G_Collinsella_HB* | Lifespan | 0.3144 | | 0.0407 | 0.022 |
| *G_Veillonella_HB* | Longevity | 0.5578 | | 0.0467 | 0.0743 |
| *G_Faecalitalea* | Healthspan | 0.2681 | | 0.0781 | 0.022 |
| *O_Lactobacillales_RNT* | Lifespan | 0.1913 | | 0.0817 | 0.0219 |
| *F_Coriobacteriaceae_RNT* | Lifespan | 0.3033 | | 0.12 | 0.022 |
| *F_Peptostreptococcaceae_HB* | Lifespan | -0.1918 | | 0.1228 | 0.022 |
| *G_Faecalitalea_RNT* | Longevity | 0.4141 | | 0.1414 | 0.0741 |
| *G_Acidaminococcus_HB* | Lifespan | 0.5402 | | 0.1515 | 0.022 |
| *O_Lactobacillales_RNT* | Longevity | 0.3009 | | 0.1553 | 0.0743 |
| *F_Streptococcaceae_RNT* | Longevity | 0.3151 | | 0.1659 | 0.0744 |
| *Div_Shannon_RNT* | Healthspan | 0.1764 | | 0.1663 | 0.022 |
| *G_Dialister_HB* | Longevity | 0.4084 | | 0.1924 | 0.0743 |
| *C_Actinobacteria_RNT* | Longevity | 0.3473 | | 0.1952 | 0.0743 |
| *G_Streptococcus_RNT* | Longevity | 0.3056 | | 0.2014 | 0.0743 |
| *G_Faecalitalea_RNT* | Lifespan | 0.1615 | | 0.2069 | 0.022 |
| *F_Desulfovibrionaceae_HB* | Healthspan | 0.2519 | | 0.2132 | 0.022 |
| *G_Intestinibacter_HB* | Lifespan | -0.2003 | | 0.2152 | 0.022 |
| *G_Subdoligranulum_HB* | Lifespan | 0.2311 | | 0.2252 | 0.022 |
| *G_Collinsella_HB* | Longevity | 0.2578 | | 0.2326 | 0.0743 |
| *G_Gemmiger_HB* | Longevity | 0.285 | | 0.234 | 0.0743 |
| *G_unclassified_C_Clostridia_RNT* | Longevity | 0.2417 | | 0.2342 | 0.0741 |
| *F_Streptococcaceae_RNT* | Lifespan | 0.1312 | | 0.2367 | 0.022 |
| *G_Bifidobacterium_RNT* | Longevity | 0.3988 | | 0.2404 | 0.0743 |
| *G_unclassified_K_Bacteria_RNT* | Lifespan | -0.108 | | 0.2468 | 0.022 |
| *G_Gemmiger_HB* | Healthspan | -0.1199 | | 0.2517 | 0.022 |
| *G_Roseburia_RNT* | Lifespan | 0.1077 | | 0.2686 | 0.022 |
| *G_unclassified_P_Proteobacteria_HB* | Longevity | -0.2822 | | 0.2712 | 0.0743 |
| *F_Acidaminococcaceae_HB* | Lifespan | 0.2563 | | 0.2729 | 0.022 |
| *F_Peptostreptococcaceae_HB* | Healthspan | -0.1292 | | 0.2787 | 0.022 |
| *G_Senegalimassilia_HB* | Healthspan | 0.2146 | | 0.2904 | 0.022 |
| *G_Streptococcus_RNT* | Lifespan | 0.123 | | 0.2937 | 0.022 |
| *G_Parasutterella_HB* | Healthspan | 0.3899 | | 0.2973 | 0.022 |
| *F_Coriobacteriaceae_RNT* | Healthspan | 0.1753 | | 0.2996 | 0.022 |
| *G_Lactobacillus_RNT* | Lifespan | 0.278 | | 0.3001 | 0.0219 |
| *G_Collinsella_HB* | Healthspan | 0.1317 | | 0.3061 | 0.022 |
| *F_Peptostreptococcaceae_HB* | Longevity | 0.302 | | 0.3183 | 0.0744 |
| *G_Hespellia_HB* | Healthspan | -0.1514 | | 0.319 | 0.022 |
| *G_Ruminococcus2_RNT* | Healthspan | 0.4483 | | 0.3194 | 0.022 |
| *G_Sporobacter_RNT* | Longevity | -0.303 | | 0.346 | 0.0743 |
| *F_Desulfovibrionaceae_HB* | Longevity | -0.2486 | | 0.3462 | 0.0743 |
| *F_Lachnospiraceae_RNT* | Longevity | 0.845 | | 0.3489 | 0.0743 |
| *C_Actinobacteria_RNT* | Lifespan | 0.1344 | | 0.3541 | 0.022 |
| *G_Blautia_RNT* | Lifespan | -0.0983 | | 0.3749 | 0.022 |
| *G_Parasutterella_HB* | Lifespan | 0.294 | | 0.3847 | 0.022 |
| *G_Acidaminococcus_HB* | Healthspan | 0.1983 | | 0.3871 | 0.022 |
| *G_Methanobrevibacter_HB* | Longevity | 0.2505 | | 0.4048 | 0.0743 |
| *G_Prevotella_HB* | Lifespan | 0.1299 | | 0.4065 | 0.022 |
| *G_Roseburia_RNT* | Healthspan | -0.0699 | | 0.4066 | 0.022 |
| *G_Victivallis_HB* | Lifespan | 0.1034 | | 0.4109 | 0.022 |
| *G_Coprobacter_HB* | Healthspan | -0.2062 | | 0.4222 | 0.022 |
| *G_Fusicatenibacter_RNT* | Longevity | 0.4899 | | 0.4261 | 0.0743 |
| *G_Ruminococcus2_RNT* | Lifespan | 0.2641 | | 0.4275 | 0.022 |
| *G_Subdoligranulum_RNT* | Longevity | 0.4383 | | 0.4321 | 0.0746 |
| *G_Eisenbergiella_HB* | Healthspan | 0.1724 | | 0.4358 | 0.022 |
| *G_Hespellia_RNT* | Longevity | 0.2106 | | 0.4417 | 0.0741 |
| *G_Butyricicoccus* | Healthspan | -0.2434 | | 0.443 | 0.022 |
| *P_Proteobacteria_RNT* | Lifespan | -0.2021 | | 0.4528 | 0.022 |
| *Div_Shannon_RNT* | Lifespan | 0.0932 | | 0.4673 | 0.022 |
| *F_Acidaminococcaceae_HB* | Healthspan | 0.1216 | | 0.4834 | 0.022 |
| *G_Methanobrevibacter_RNT* | Lifespan | 0.1566 | | 0.4861 | 0.0219 |
| *G_Parasutterella_HB* | Longevity | 0.3573 | | 0.4863 | 0.0743 |
| *G_Blautia_RNT* | Longevity | 0.1384 | | 0.4899 | 0.0743 |
| *G_Collinsella_RNT* | Lifespan | 0.2261 | | 0.4967 | 0.022 |
| *G_Prevotella_HB* | Healthspan | 0.1152 | | 0.4987 | 0.022 |
| *G_Blautia_RNT* | Healthspan | 0.067 | | 0.5005 | 0.022 |
| *Div_NumberGenera_RNT* | Healthspan | 0.0848 | | 0.5023 | 0.022 |
| *G_Alloprevotella_HB* | Lifespan | 0.1001 | | 0.5132 | 0.022 |
| *G_Prevotella_HB* | Longevity | -0.2663 | | 0.5144 | 0.0743 |
| *G_unclassified_F_Porphyromonadaceae_RNT* | Longevity | 0.1712 | | 0.5266 | 0.0745 |
| *G_unclassified_F_Erysipelotrichaceae_HB* | Lifespan | 0.0944 | | 0.5318 | 0.022 |
| *G_Subdoligranulum_RNT* | Healthspan | -0.1482 | | 0.5385 | 0.0219 |
| *P_Lentisphaerae_HB* | Lifespan | 0.0925 | | 0.5398 | 0.022 |
| *G_unclassified_P_Bacteroidetes_HB* | Lifespan | -0.2598 | | 0.5411 | 0.022 |
| *G_Clostridium_IV_RNT* | Longevity | -0.2477 | | 0.5484 | 0.0743 |
| *G_unclassified_K_Bacteria_HB* | Healthspan | 0.088 | | 0.5506 | 0.022 |
| *G_Victivallis_HB* | Longevity | 0.1407 | | 0.5606 | 0.0743 |
| *C_Gammaproteobacteria_RNT* | Longevity | -0.1949 | | 0.5626 | 0.0744 |
| *G_unclassified_P_Proteobacteria_HB* | Healthspan | -0.0668 | | 0.5636 | 0.022 |
| *P_Proteobacteria_RNT* | Longevity | -0.4858 | | 0.5751 | 0.0743 |
| *G_unclassified_C_Clostridia_HB* | Healthspan | 0.1003 | | 0.5753 | 0.022 |
| *G_Fusicatenibacter* | Healthspan | -0.177 | | 0.5788 | 0.022 |
| *G_Clostridium_sensu_stricto_RNT* | Longevity | -0.5109 | | 0.5789 | 0.0743 |
| *G_Hespellia_RNT* | Lifespan | 0.0767 | | 0.5856 | 0.0219 |
| *G_Sporobacter_RNT* | Lifespan | -0.0833 | | 0.59 | 0.0219 |
| *G_Acidaminococcus_HB* | Longevity | 0.2045 | | 0.5993 | 0.0743 |
| *G_unclassified_K_Bacteria_RNT* | Healthspan | -0.0489 | | 0.5995 | 0.022 |
| *G_Clostridium_IV_RNT* | Healthspan | 0.0973 | | 0.5998 | 0.022 |
| *G_Collinsella_RNT* | Healthspan | 0.1375 | | 0.6039 | 0.022 |
| *G_unclassified_F_Erysipelotrichaceae_HB* | Longevity | -0.1431 | | 0.6059 | 0.0743 |
| *Div_Shannon_RNT* | Longevity | 0.1254 | | 0.6082 | 0.0743 |
| *G_unclassified_O_Clostridiales_RNT* | Lifespan | -0.0684 | | 0.6088 | 0.022 |
| *Div_Chao1_RNT* | Lifespan | 0.066 | | 0.6089 | 0.022 |
| *G_Butyrivibrio_RNT* | Lifespan | -0.1441 | | 0.6139 | 0.022 |
| *G_Odoribacter_HB* | Longevity | -1.0562 | | 0.6146 | 0.0745 |
| *G_Subdoligranulum_HB* | Longevity | -0.1607 | | 0.6151 | 0.0743 |
| *G_unclassified_F_Porphyromonadaceae_RNT* | Healthspan | 0.1269 | | 0.6258 | 0.0219 |
| *O_Lactobacillales_RNT* | Healthspan | 0.0487 | | 0.6267 | 0.0219 |
| *G_Dorea_RNT* | Longevity | 0.1321 | | 0.6286 | 0.0743 |
| *C_Actinobacteria_RNT* | Healthspan | 0.0563 | | 0.6295 | 0.022 |
| *F_Acidaminococcaceae_HB* | Longevity | -0.1662 | | 0.6371 | 0.0743 |
| *G_Sporobacter_RNT* | Healthspan | -0.0655 | | 0.6394 | 0.0219 |
| *G_Senegalimassilia_HB* | Longevity | -0.1747 | | 0.6424 | 0.0743 |
| *Div_NumberGenera_RNT* | Lifespan | 0.0557 | | 0.6424 | 0.022 |
| *G_unclassified_F_Porphyromonadaceae_HB* | Healthspan | 0.0588 | | 0.6427 | 0.022 |
| *G_Hespellia_HB* | Longevity | 0.1444 | | 0.645 | 0.0743 |
| *G_unclassified_F_Porphyromonadaceae_RNT* | Lifespan | 0.1034 | | 0.6477 | 0.0219 |
| *G_Odoribacter_HB* | Lifespan | -0.1767 | | 0.6495 | 0.022 |
| *G_Subdoligranulum_HB* | Healthspan | 0.0635 | | 0.6541 | 0.022 |
| *G_unclassified_F_Erysipelotrichaceae_HB* | Healthspan | 0.0672 | | 0.6592 | 0.022 |
| *G_Akkermansia_HB* | Lifespan | 0.1132 | | 0.6593 | 0.022 |
| *G_unclassified_F_Ruminococcaceae_RNT* | Lifespan | -0.0817 | | 0.6615 | 0.022 |
| *C_Deltaproteobacteria_HB* | Healthspan | 0.5451 | | 0.6635 | 0.022 |
| *G_unclassified_P_Bacteroidetes_HB* | Longevity | 0.4961 | | 0.6678 | 0.0743 |
| *F_Enterococcaceae_HB* | Longevity | 0.2631 | | 0.668 | 0.0743 |
| *G_unclassified_O_Clostridiales_RNT* | Longevity | -0.1317 | | 0.6684 | 0.0743 |
| *G_Sporobacter_HB* | Longevity | 0.0822 | | 0.6711 | 0.0743 |
| *G_unclassified_C_Clostridia_HB* | Longevity | -0.1732 | | 0.6748 | 0.0743 |
| *G_Methanobrevibacter_HB* | Lifespan | -0.0687 | | 0.6759 | 0.022 |
| *G_unclassified_F_Ruminococcaceae_RNT* | Healthspan | 0.0683 | | 0.6781 | 0.022 |
| *G_Desulfovibrio_RNT* | Lifespan | 0.0822 | | 0.6829 | 0.0219 |
| *G_Butyricicoccus_RNT* | Longevity | 0.1786 | | 0.6834 | 0.0743 |
| *G_Butyrivibrio_RNT* | Longevity | 0.3604 | | 0.6924 | 0.0746 |
| *F_Streptococcaceae_RNT* | Healthspan | 0.0425 | | 0.6934 | 0.022 |
| *G_unclassified_C_Clostridia_HB* | Lifespan | -0.0696 | | 0.6985 | 0.022 |
| *G_unclassified_P_Proteobacteria_HB* | Lifespan | -0.0435 | | 0.7028 | 0.022 |
| *Div_Chao1_RNT* | Healthspan | 0.051 | | 0.7046 | 0.022 |
| *G_Hespellia_HB* | Lifespan | 0.0543 | | 0.7152 | 0.022 |
| *G_Butyricicoccus_RNT* | Lifespan | -0.0785 | | 0.7154 | 0.022 |
| *G_unclassified_C_Clostridia_RNT* | Healthspan | -0.0405 | | 0.7195 | 0.0219 |
| *G_unclassified_F_Ruminococcaceae_RNT* | Longevity | 0.1318 | | 0.7198 | 0.0743 |
| *G_Streptococcus_RNT* | Healthspan | 0.0408 | | 0.7217 | 0.022 |
| *G_Oscillibacter_RNT* | Healthspan | 0.1241 | | 0.7238 | 0.022 |
| *G_Alloprevotella_HB* | Healthspan | -0.0573 | | 0.7264 | 0.022 |
| *G_Odoribacter_RNT* | Lifespan | -0.2906 | | 0.7317 | 0.022 |
| *P_Lentisphaerae_HB* | Healthspan | 0.049 | | 0.7318 | 0.022 |
| *G_Coprococcus_RNT* | Longevity | 0.5726 | | 0.7338 | 0.0742 |
| *G_unclassified_O_Clostridiales_RNT* | Healthspan | 0.0422 | | 0.7351 | 0.022 |
| *F_Coriobacteriaceae_RNT* | Longevity | 0.1026 | | 0.7357 | 0.0743 |
| *G_Ruminococcus_HB* | Lifespan | -0.042 | | 0.7375 | 0.022 |
| *G_unclassified_P_Bacteroidetes_HB* | Healthspan | 0.0825 | | 0.7409 | 0.022 |
| *G_Odoribacter_RNT* | Healthspan | 0.1873 | | 0.7427 | 0.022 |
| *F_Desulfovibrionaceae_RNT* | Lifespan | -0.0717 | | 0.7439 | 0.0219 |
| *G_unclassified_C_Clostridia_RNT* | Lifespan | -0.0362 | | 0.7452 | 0.0219 |
| *G_Lactobacillus_HB* | Longevity | -0.6014 | | 0.7509 | 0.0743 |
| *C_Gammaproteobacteria_RNT* | Lifespan | -0.0431 | | 0.7528 | 0.022 |
| *G_Oscillibacter_RNT* | Lifespan | 0.1084 | | 0.7531 | 0.022 |
| *G_Hespellia_RNT* | Healthspan | 0.0386 | | 0.7572 | 0.0219 |
| *F_Lachnospiraceae_RNT* | Healthspan | 0.0549 | | 0.7588 | 0.022 |
| *G_Ruminococcus_HB* | Longevity | -0.0879 | | 0.762 | 0.0745 |
| *C_Gammaproteobacteria_RNT* | Healthspan | -0.038 | | 0.7667 | 0.022 |
| *G_Sporobacter_HB* | Lifespan | -0.0269 | | 0.767 | 0.022 |
| *G_Lactobacillus_RNT* | Longevity | 0.1129 | | 0.77 | 0.0742 |
| *G_Clostridium_IV_RNT* | Lifespan | 0.0546 | | 0.7752 | 0.022 |
| *G_Bifidobacterium_RNT* | Lifespan | 0.0442 | | 0.7801 | 0.0219 |
| *G_Eisenbergiella_HB* | Lifespan | -0.0482 | | 0.7851 | 0.022 |
| *G_Senegalimassilia_HB* | Lifespan | 0.047 | | 0.7877 | 0.022 |
| *G_Collinsella_RNT* | Longevity | -0.1191 | | 0.7892 | 0.0742 |
| *G_unclassified_K_Bacteria_HB* | Lifespan | -0.0399 | | 0.7939 | 0.022 |
| *G_Desulfovibrio_RNT* | Healthspan | 0.0446 | | 0.7939 | 0.0219 |
| *G_Desulfovibrio_RNT* | Longevity | 0.0764 | | 0.7972 | 0.0741 |
| *F_Lachnospiraceae_RNT* | Lifespan | 0.0491 | | 0.7997 | 0.022 |
| *G_Odoribacter_HB* | Healthspan | -0.067 | | 0.8127 | 0.022 |
| *G_Gemmiger_HB* | Lifespan | 0.0257 | | 0.8159 | 0.022 |
| *G_Subdoligranulum_RNT* | Lifespan | 0.046 | | 0.8181 | 0.0219 |
| *G_Akkermansia_HB* | Healthspan | 0.416 | | 0.8245 | 0.022 |
| *Div_Chao1_RNT* | Longevity | -0.0527 | | 0.8255 | 0.0743 |
| *G_Eisenbergiella_HB* | Longevity | 0.0883 | | 0.8291 | 0.0743 |
| *G_Veillonella_HB* | Lifespan | 0.0215 | | 0.8346 | 0.022 |
| *G_Dialister_HB* | Healthspan | 0.0242 | | 0.835 | 0.022 |
| *G_Coprococcus_RNT* | Healthspan | -0.0506 | | 0.8356 | 0.0219 |
| *G_Dorea_RNT* | Lifespan | 0.0275 | | 0.8389 | 0.022 |
| *G_Butyrivibrio_RNT* | Healthspan | 0.0381 | | 0.8438 | 0.022 |
| *G_Dialister_HB* | Lifespan | -0.0214 | | 0.8479 | 0.022 |
| *G_Coprobacter_HB* | Lifespan | 0.0365 | | 0.8482 | 0.022 |
| *C_Deltaproteobacteria_HB* | Longevity | -0.0771 | | 0.8503 | 0.0743 |
| *G_Veillonella_HB* | Healthspan | 0.0186 | | 0.8517 | 0.022 |
| *G_Butyrivibrio_HB* | Longevity | 0.1185 | | 0.8594 | 0.0743 |
| *G_unclassified_K_Bacteria_HB* | Longevity | 0.0522 | | 0.8607 | 0.0743 |
| *G_unclassified_F_Porphyromonadaceae_HB* | Lifespan | 0.0211 | | 0.8619 | 0.022 |
| *P_Proteobacteria_RNT* | Healthspan | -0.0346 | | 0.8638 | 0.022 |
| *G_unclassified_F_Porphyromonadaceae_HB* | Longevity | 0.037 | | 0.8663 | 0.0743 |
| *G_Coprococcus_RNT* | Lifespan | -0.0372 | | 0.8819 | 0.0219 |
| *P_Lentisphaerae_HB* | Longevity | -0.04 | | 0.8832 | 0.0743 |
| *G_Methanobrevibacter_RNT* | Healthspan | 0.0236 | | 0.8962 | 0.0219 |
| *G_Fusicatenibacter_RNT* | Lifespan | -0.0252 | | 0.9123 | 0.022 |
| *G_Lactobacillus_RNT* | Healthspan | -0.0175 | | 0.9146 | 0.0219 |
| *G_Methanobrevibacter_RNT* | Longevity | -0.0378 | | 0.9175 | 0.0743 |
| *G_Dorea_RNT* | Healthspan | -0.013 | | 0.9189 | 0.022 |
| *G_Alloprevotella_HB* | Longevity | 0.0263 | | 0.9196 | 0.0743 |
| *G_Intestinibacter_HB* | Healthspan | -0.0141 | | 0.9199 | 0.022 |
| *F_Desulfovibrionaceae_HB* | Lifespan | -0.0131 | | 0.927 | 0.022 |
| *G_Coprobacter_HB* | Longevity | 0.03 | | 0.9361 | 0.0743 |
| *F_Desulfovibrionaceae_RNT* | Longevity | -0.0237 | | 0.9454 | 0.0743 |
| *G_unclassified_K_Bacteria_RNT* | Longevity | 0.0131 | | 0.9466 | 0.0744 |
| *Div_NumberGenera_RNT* | Longevity | -0.0132 | | 0.9517 | 0.0743 |
| *F_Desulfovibrionaceae_RNT* | Healthspan | -0.0112 | | 0.9529 | 0.0219 |
| *G_Victivallis_HB* | Healthspan | 0.0063 | | 0.9577 | 0.022 |
| *G_Methanobrevibacter_HB* | Healthspan | 0.0075 | | 0.9588 | 0.022 |
| *C_Deltaproteobacteria_HB* | Lifespan | -0.0133 | | 0.9617 | 0.022 |
| *G_Intestinibacter_HB* | Longevity | -0.0054 | | 0.9852 | 0.0743 |
| *G_Coprococcus_HB* | Longevity | 0.0082 | | 0.9854 | 0.0743 |
| *G_Ruminococcus_HB* | Healthspan | 0.0019 | | 0.9882 | 0.022 |
| *G_Bifidobacterium_RNT* | Healthspan | 0.0018 | | 0.9891 | 0.0219 |

***Note*：**C, class; O, order; F, family; G, genus; RNT, rank-normal transformation; HB, hurdle binary.
