# Supplementary material for: Association between gut microbiota and longevity: a genetic correlation and mendelian randomization study
Source: BMC Microbiol. 2022 Dec 13;22:302. doi: 10.1186/s12866-022-02703-x (PMC9746102; doi:10.1186/s12866-022-02703-x)
Supplement: Supplementary file 3 — Additional file 3: Supplementary Table 3. The MR analysis results of gut microbiota on longevity-related traits. [file 12866_2022_2703_MOESM3_ESM.docx]

**Supplementary table 3**. The MR analysis results of gut microbiota on longevity-related traits

| **MR** | | | | | | | **Heterogeneity** | **Pleiotropy** |
| --- | --- | --- | --- | --- | --- | --- | --- | --- |
| **Exposure** | **Outcome** | **Method** | **Nsnp** | **b** | **Se** | ***P*** | ***P*** | ***P*** |
| *G_Collinsella_HB* | Parental longevity (father's age at death) | MR Egger | 13 | 8.38E-04 | 1.42E-03 | 5.66E-01 | 0.4302 | 0.7567 |
|  |  | Weighted median | 13 | 1.79E-03 | 8.48E-04 | 3.52E-02 | - | - |
|  |  | Inverse variance weighted | 13 | 1.23E-03 | 6.86E-04 | 7.29E-02 | 0.5068 | - |
|  |  | Simple mode | 13 | -5.42E-05 | 2.06E-03 | 9.79E-01 | - | - |
|  |  | Weighted mode | 13 | 1.52E-03 | 7.95E-04 | 8.00E-02 | - | - |
|  | Parental longevity (father's attained age) | MR Egger | 14 | -2.10E-04 | 9.34E-04 | 8.26E-01 | 0.4904 | 0.8004 |
|  |  | Weighted median | 14 | -7.25E-04 | 7.76E-04 | 3.50E-01 | - | - |
|  |  | Inverse variance weighted | 14 | -3.98E-04 | 5.83E-04 | 4.95E-01 | 0.5672 | - |
|  |  | Simple mode | 14 | -3.36E-05 | 1.29E-03 | 9.80E-01 | - | - |
|  |  | Weighted mode | 14 | -6.19E-04 | 8.55E-04 | 4.82E-01 | - | - |
|  | Parental extreme longevity (95 years and older) | MR Egger | 13 | 1.09E-04 | 2.81E-04 | 7.05E-01 | 0.3164 | 0.4863 |
|  |  | Weighted median | 13 | -6.00E-05 | 1.53E-04 | 6.95E-01 | - | - |
|  |  | Inverse variance weighted | 13 | -6.76E-05 | 1.35E-04 | 6.16E-01 | 0.3509 | - |
|  |  | Simple mode | 13 | 4.52E-05 | 3.64E-04 | 9.03E-01 | - | - |
|  |  | Weighted mode | 13 | -6.70E-05 | 1.45E-04 | 6.53E-01 | - | - |
|  | Parental longevity (both parents in top 10%) | MR Egger | 14 | 2.71E-04 | 1.09E-03 | 8.08E-01 | 0.0689 | 0.8919 |
|  |  | Weighted median | 14 | -4.78E-05 | 8.15E-04 | 9.53E-01 | - | - |
|  |  | Inverse variance weighted | 14 | 3.89E-04 | 6.57E-04 | 5.53E-01 | 0.0968 | - |
|  |  | Simple mode | 14 | -8.68E-04 | 1.47E-03 | 5.66E-01 | - | - |
|  |  | Weighted mode | 14 | 1.15E-03 | 1.09E-03 | 3.11E-01 | - | - |
|  | Parental longevity (combined parental age at death) | MR Egger | 13 | -1.74E-03 | 2.32E-03 | 4.69E-01 | 0.8371 | 0.3944 |
|  |  | Weighted median | 13 | -1.82E-05 | 1.30E-03 | 9.89E-01 | - | - |
|  |  | Inverse variance weighted | 13 | 5.53E-05 | 1.13E-03 | 9.61E-01 | 0.8373 | - |
|  |  | Simple mode | 13 | -4.28E-05 | 2.83E-03 | 9.88E-01 | - | - |
|  |  | Weighted mode | 13 | 1.39E-04 | 1.28E-03 | 9.15E-01 | - | - |
|  | Parental longevity (combined parental attained age, Martingale residuals) | MR Egger | 14 | -4.24E-04 | 1.18E-03 | 7.25E-01 | 0.1150 | 0.9785 |
|  |  | Weighted median | 14 | -1.44E-03 | 8.96E-04 | 1.08E-01 | - | - |
|  |  | Inverse variance weighted | 14 | -3.99E-04 | 7.07E-04 | 5.73E-01 | 0.1566 | - |
|  |  | Simple mode | 14 | -1.72E-03 | 1.60E-03 | 3.02E-01 | - | - |
|  |  | Weighted mode | 14 | -1.34E-03 | 1.05E-03 | 2.25E-01 | - | - |
|  | Parental longevity (combined parental age at death) | MR Egger | 14 | 6.51E-04 | 1.59E-03 | 6.90E-01 | 0.2173 | 0.9221 |
|  |  | Weighted median | 14 | 7.46E-04 | 1.18E-03 | 5.28E-01 | - | - |
|  |  | Inverse variance weighted | 14 | 5.27E-04 | 9.56E-04 | 5.82E-01 | 0.2788 | - |
|  |  | Simple mode | 14 | 7.68E-04 | 1.98E-03 | 7.04E-01 | - | - |
|  |  | Weighted mode | 14 | 9.68E-04 | 1.34E-03 | 4.83E-01 | - | - |
|  | Parental longevity (father's age at death) | MR Egger | 14 | -4.97E-04 | 1.19E-03 | 6.84E-01 | 0.3514 | 0.9623 |
|  |  | Weighted median | 14 | 1.13E-04 | 1.02E-03 | 9.12E-01 | - | - |
|  |  | Inverse variance weighted | 14 | -5.42E-04 | 7.17E-04 | 4.49E-01 | 0.4287 | - |
|  |  | Simple mode | 14 | 8.94E-04 | 1.76E-03 | 6.21E-01 | - | - |
|  |  | Weighted mode | 14 | 9.35E-04 | 1.25E-03 | 4.68E-01 | - | - |
|  | Parental longevity (mother's attained age) | MR Egger | 14 | 4.09E-04 | 1.03E-03 | 6.97E-01 | 0.2785 | 0.8896 |
|  |  | Weighted median | 14 | -7.71E-04 | 8.59E-04 | 3.69E-01 | - | - |
|  |  | Inverse variance weighted | 14 | 2.95E-04 | 6.17E-04 | 6.32E-01 | 0.3475 | - |
|  |  | Simple mode | 14 | -1.08E-03 | 1.37E-03 | 4.43E-01 | - | - |
|  |  | Weighted mode | 14 | -9.62E-04 | 9.44E-04 | 3.27E-01 | - | - |
|  | Parental longevity (mother's age at death) | MR Egger | 14 | 8.20E-04 | 1.47E-03 | 5.86E-01 | 0.1072 | 0.6779 |
|  |  | Weighted median | 14 | 1.18E-03 | 1.09E-03 | 2.78E-01 | - | - |
|  |  | Inverse variance weighted | 14 | 3.33E-04 | 8.87E-04 | 7.08E-01 | 0.1373 | - |
|  |  | Simple mode | 14 | 8.28E-04 | 1.53E-03 | 5.97E-01 | - | - |
|  |  | Weighted mode | 14 | 1.42E-03 | 1.12E-03 | 2.27E-01 | - | - |
|  | Lifespan | MR Egger | 16 | -2.33E-04 | 1.59E-03 | 8.86E-01 | 0.4621 | 0.5915 |
|  |  | Weighted median | 16 | -4.12E-04 | 1.41E-03 | 7.71E-01 | - | - |
|  |  | Inverse variance weighted | 16 | -9.25E-04 | 9.76E-04 | 3.43E-01 | 0.5152 | - |
|  |  | Simple mode | 16 | -5.21E-03 | 2.65E-03 | 6.78E-02 | - | - |
|  |  | Weighted mode | 16 | 5.31E-04 | 1.71E-03 | 7.61E-01 | - | - |
|  | Healthspan | MR Egger | 15 | -1.69E-04 | 2.12E-03 | 9.38E-01 | 0.3556 | 0.7374 |
|  |  | Weighted median | 15 | 2.73E-04 | 1.80E-03 | 8.80E-01 | - | - |
|  |  | Inverse variance weighted | 15 | -7.41E-04 | 1.26E-03 | 5.55E-01 | 0.4210 | - |
|  |  | Simple mode | 15 | 2.13E-04 | 2.62E-03 | 9.36E-01 | - | - |
|  |  | Weighted mode | 15 | 7.38E-04 | 1.98E-03 | 7.15E-01 | - | - |
|  | Longevity | MR Egger | 7 | -1.83E-02 | 2.02E-02 | 4.06E-01 | 0.8077 | 0.6931 |
|  |  | Weighted median | 7 | -7.25E-03 | 1.48E-02 | 6.23E-01 | - | - |
|  |  | Inverse variance weighted | 7 | -1.15E-02 | 1.19E-02 | 3.33E-01 | 0.8723 | - |
|  |  | Simple mode | 7 | -1.20E-03 | 2.22E-02 | 9.59E-01 | - | - |
|  |  | Weighted mode | 7 | -4.52E-03 | 1.98E-02 | 8.27E-01 | - | - |
| *G_Sporobacter_HB* | Parental longevity (mother's attained age) | MR Egger | 20 | 9.21E-04 | 1.20E-03 | 4.54E-01 | 0.3152 | 0.5250 |
|  |  | Weighted median | 20 | 5.48E-04 | 6.32E-04 | 3.86E-01 | - | - |
|  |  | Inverse variance weighted | 20 | 2.04E-04 | 4.65E-04 | 6.61E-01 | 0.3484 | - |
|  |  | Simple mode | 20 | 3.49E-04 | 1.04E-03 | 7.42E-01 | - | - |
|  |  | Weighted mode | 20 | 6.68E-04 | 7.81E-04 | 4.03E-01 | - | - |
|  | Parental longevity (father's age at death) | MR Egger | 16 | 3.18E-04 | 2.62E-03 | 9.05E-01 | 0.2552 | 0.8345 |
|  |  | Weighted median | 16 | 1.05E-03 | 1.01E-03 | 2.95E-01 | - | - |
|  |  | Inverse variance weighted | 16 | 8.49E-04 | 7.74E-04 | 2.73E-01 | 0.3144 | - |
|  |  | Simple mode | 16 | 1.22E-03 | 1.74E-03 | 4.93E-01 | - | - |
|  |  | Weighted mode | 16 | 7.86E-04 | 1.04E-03 | 4.59E-01 | - | - |
|  | Parental longevity (father's attained age) | MR Egger | 20 | 9.39E-04 | 1.38E-03 | 5.05E-01 | 0.0805 | 0.2587 |
|  |  | Weighted median | 20 | 4.26E-04 | 6.43E-04 | 5.08E-01 | - | - |
|  |  | Inverse variance weighted | 20 | -5.40E-04 | 5.46E-04 | 3.23E-01 | 0.0667 | - |
|  |  | Simple mode | 20 | 3.80E-05 | 1.05E-03 | 9.72E-01 | - | - |
|  |  | Weighted mode | 20 | 4.59E-04 | 7.30E-04 | 5.37E-01 | - | - |
|  | Parental longevity (combined parental age at death) | MR Egger | 20 | -2.39E-03 | 1.70E-03 | 1.75E-01 | 0.6605 | 0.2953 |
|  |  | Weighted median | 20 | -8.92E-04 | 9.72E-04 | 3.59E-01 | - | - |
|  |  | Inverse variance weighted | 20 | -7.13E-04 | 6.66E-04 | 2.84E-01 | 0.6450 | - |
|  |  | Simple mode | 20 | -2.14E-03 | 1.76E-03 | 2.37E-01 | - | - |
|  |  | Weighted mode | 20 | -2.57E-03 | 1.40E-03 | 8.30E-02 | - | - |
|  | Parental longevity (combined parental age at death) | MR Egger | 16 | 5.46E-03 | 3.91E-03 | 1.84E-01 | 0.5973 | 0.3418 |
|  |  | Weighted median | 16 | 1.12E-03 | 1.67E-03 | 5.04E-01 | - | - |
|  |  | Inverse variance weighted | 16 | 1.80E-03 | 1.19E-03 | 1.32E-01 | 0.5961 | - |
|  |  | Simple mode | 16 | 2.45E-03 | 3.00E-03 | 4.26E-01 | - | - |
|  |  | Weighted mode | 16 | 1.24E-03 | 1.67E-03 | 4.69E-01 | - | - |
|  | Parental longevity (father's age at death) | MR Egger | 20 | 3.80E-04 | 1.60E-03 | 8.15E-01 | 0.1416 | 0.8488 |
|  |  | Weighted median | 20 | -2.53E-04 | 8.39E-04 | 7.63E-01 | - | - |
|  |  | Inverse variance weighted | 20 | 9.58E-05 | 6.12E-04 | 8.76E-01 | 0.1786 | - |
|  |  | Simple mode | 20 | -1.33E-03 | 1.61E-03 | 4.21E-01 | - | - |
|  |  | Weighted mode | 20 | 1.88E-03 | 1.32E-03 | 1.71E-01 | - | - |
|  | Parental longevity (combined parental attained age, Martingale residuals) | MR Egger | 20 | 1.10E-03 | 1.73E-03 | 5.34E-01 | 0.0019 | 0.4459 |
|  |  | Weighted median | 20 | 6.94E-04 | 6.50E-04 | 2.85E-01 | - | - |
|  |  | Inverse variance weighted | 20 | -1.43E-04 | 6.74E-04 | 8.32E-01 | 0.0020 | - |
|  |  | Simple mode | 20 | 1.33E-03 | 9.06E-04 | 1.57E-01 | - | - |
|  |  | Weighted mode | 20 | 8.03E-04 | 7.33E-04 | 2.87E-01 | - | - |
|  | Parental longevity (both parents in top 10%) | MR Egger | 20 | -6.36E-04 | 1.13E-03 | 5.81E-01 | 0.2358 | 0.5854 |
|  |  | Weighted median | 20 | 4.30E-05 | 6.32E-04 | 9.46E-01 | - | - |
|  |  | Inverse variance weighted | 20 | -5.78E-05 | 4.36E-04 | 8.94E-01 | 0.2701 | - |
|  |  | Simple mode | 20 | 8.43E-04 | 1.21E-03 | 4.93E-01 | - | - |
|  |  | Weighted mode | 20 | 4.07E-05 | 7.89E-04 | 9.59E-01 | - | - |
|  | Parental extreme longevity (95 years and older) | MR Egger | 16 | -1.98E-05 | 4.48E-04 | 9.65E-01 | 0.5501 | 0.8696 |
|  |  | Weighted median | 16 | -4.39E-05 | 1.92E-04 | 8.19E-01 | - | - |
|  |  | Inverse variance weighted | 16 | 5.16E-05 | 1.36E-04 | 7.04E-01 | 0.6231 | - |
|  |  | Simple mode | 16 | -6.31E-05 | 2.98E-04 | 8.36E-01 | - | - |
|  |  | Weighted mode | 16 | -7.19E-05 | 1.77E-04 | 6.91E-01 | - | - |
|  | Parental longevity (mother's age at death) | MR Egger | 20 | -4.73E-04 | 1.43E-03 | 7.45E-01 | 0.9959 | 0.9538 |
|  |  | Weighted median | 20 | 1.01E-04 | 7.71E-04 | 8.96E-01 | - | - |
|  |  | Inverse variance weighted | 20 | -3.96E-04 | 5.62E-04 | 4.81E-01 | 0.9977 | - |
|  |  | Simple mode | 20 | -1.25E-03 | 1.18E-03 | 3.03E-01 | - | - |
|  |  | Weighted mode | 20 | 8.87E-05 | 9.96E-04 | 9.30E-01 | - | - |
|  | Lifespan | MR Egger | 20 | -6.81E-04 | 2.12E-03 | 7.52E-01 | 0.7960 | 0.7014 |
|  |  | Weighted median | 20 | -7.63E-04 | 1.13E-03 | 5.00E-01 | - | - |
|  |  | Inverse variance weighted | 20 | 8.50E-05 | 8.03E-04 | 9.16E-01 | 0.8346 | - |
|  |  | Simple mode | 20 | -5.11E-04 | 1.94E-03 | 7.95E-01 | - | - |
|  |  | Weighted mode | 20 | -6.42E-04 | 1.29E-03 | 6.24E-01 | - | - |
|  | Healthspan | MR Egger | 15 | -4.17E-03 | 2.71E-03 | 1.48E-01 | 0.3045 | 0.1366 |
|  |  | Weighted median | 15 | -1.54E-04 | 1.61E-03 | 9.24E-01 | - | - |
|  |  | Inverse variance weighted | 15 | -2.81E-04 | 1.22E-03 | 8.18E-01 | 0.2086 | - |
|  |  | Simple mode | 15 | -7.69E-05 | 2.14E-03 | 9.72E-01 | - | - |
|  |  | Weighted mode | 15 | -4.28E-04 | 1.72E-03 | 8.07E-01 | - | - |
|  | Longevity | MR Egger | 5 | -3.33E-02 | 4.42E-02 | 5.06E-01 | 0.3234 | 0.5356 |
|  |  | Weighted median | 5 | -4.48E-03 | 5.18E-03 | 3.87E-01 | - | - |
|  |  | Inverse variance weighted | 5 | -2.64E-03 | 4.87E-03 | 5.88E-01 | 0.4001 | - |
|  |  | Simple mode | 5 | -4.81E-03 | 1.34E-02 | 7.37E-01 | - | - |
|  |  | Weighted mode | 5 | -4.65E-03 | 4.88E-03 | 3.95E-01 | - | - |
| *G_Veillonella_HB* | Parental longevity (mother's attained age) | Wald ratio | 1 | 7.27E-03 | 2.97E-03 | 1.43E-02 | - | - |
|  | Lifespan | Wald ratio | 1 | -2.43E-02 | 1.00E-02 | 1.52E-02 | - | - |
|  | Healthspan | Wald ratio | 1 | -2.59E-02 | 1.17E-02 | 2.67E-02 | - | - |
|  | Parental longevity (combined parental attained age, Martingale residuals) | Wald ratio | 1 | 6.36E-03 | 3.04E-03 | 3.62E-02 | - | - |
|  | Parental longevity (both parents in top 10%) | Wald ratio | 1 | -4.34E-03 | 2.69E-03 | 1.07E-01 | - | - |
|  | Parental longevity (father's attained age) | Wald ratio | 1 | 3.90E-03 | 2.96E-03 | 1.87E-01 | - | - |
|  | Longevity | Wald ratio | 1 | -7.89E-02 | 7.23E-02 | 2.75E-01 | - | - |
|  | Parental extreme longevity (95 years and older) | Wald ratio | 1 | -1.14E-03 | 1.15E-03 | 3.23E-01 | - | - |
|  | Parental longevity (father's age at death) | Wald ratio | 1 | -2.02E-03 | 3.61E-03 | 5.76E-01 | - | - |
|  | Parental longevity (father's age at death) | Wald ratio | 1 | 3.35E-03 | 6.13E-03 | 5.85E-01 | - | - |
|  | Parental longevity (combined parental age at death) | Wald ratio | 1 | 1.67E-03 | 1.00E-02 | 8.67E-01 | - | - |
|  | Parental longevity (combined parental age at death) | Wald ratio | 1 | 2.56E-04 | 4.48E-03 | 9.54E-01 | - | - |
|  | Parental longevity (mother's age at death) | Wald ratio | 1 | 1.63E-04 | 3.78E-03 | 9.66E-01 | - | - |

***Note*:** G, genus; RNT, rank-normal transformation; HB, hurdle binary; b means the estimated causal effect.
