# Supplementary material for: Association between gut microbiota and longevity: a genetic correlation and mendelian randomization study
Source: BMC Microbiol. 2022 Dec 13;22:302. doi: 10.1186/s12866-022-02703-x (PMC9746102; doi:10.1186/s12866-022-02703-x)
Supplement: Supplementary file 4 — Additional file 4: Supplementary Table 4. The reverse MR analysis results of longevity-related traits on gut microbiota. [file 12866_2022_2703_MOESM4_ESM.docx]

**Supplementary table 4.** The reverse MR analysis results of longevity-related traits on gut microbiota

| **MR** | | | | | | | **Heterogeneity** | **Pleiotropy** |
| --- | --- | --- | --- | --- | --- | --- | --- | --- |
| **Exposure** | **Outcome** | **Method** | **N_SNP_** | **b** | **Se** | ***P*** | ***P*** | ***P*** |
| Longevity | *G_Collinsella_HB* | MR Egger | 42 | -2.04E-01 | 1.58E-01 | 2.05E-01 | 1.0000 | 0.4595 |
|  |  | Weighted median | 42 | -3.06E-01 | 9.34E-02 | 1.05E-03 | - | - |
|  |  | Inverse variance weighted | 42 | -3.10E-01 | 7.14E-02 | 1.43E-05 | 1.0000 | - |
|  |  | Simple mode | 42 | -3.14E-01 | 1.59E-01 | 5.42E-02 | - | - |
|  |  | Weighted mode | 42 | -2.83E-01 | 1.36E-01 | 4.41E-02 | - | - |
| Healthspan |  | MR Egger | 331 | -1.19E+00 | 7.40E-01 | 1.07E-01 | 1.0000 | 0.1078 |
|  |  | Weighted median | 331 | -8.63E-02 | 2.39E-01 | 7.18E-01 |  | - |
|  |  | Inverse variance weighted | 331 | -3.66E-02 | 1.79E-01 | 8.39E-01 | 1.0000 | - |
|  |  | Simple mode | 331 | 1.31E+00 | 7.64E-01 | 8.61E-02 |  | - |
|  |  | Weighted mode | 331 | -1.40E+00 | 6.94E-01 | 4.40E-02 |  | - |
| Lifespan |  | MR Egger | 431 | -2.20E+00 | 4.46E-01 | 1.25E-06 | 1.0000 | 0.0001 |
|  |  | Weighted median | 431 | -1.36E+00 | 2.40E-01 | 1.62E-08 |  | - |
|  |  | Inverse variance weighted | 431 | -5.90E-01 | 1.65E-01 | 3.46E-04 | 1.0000 | - |
|  |  | Simple mode | 431 | -1.53E+00 | 5.99E-01 | 1.11E-02 |  | - |
|  |  | Weighted mode | 431 | -1.53E+00 | 4.47E-01 | 6.84E-04 |  | - |
| Parental longevity (mother's attained age) |  | MR Egger | 4 | 7.25E+00 | 7.18E+00 | 4.19E-01 | 0.3707 | 0.4890 |
|  |  | Weighted median | 4 | 1.52E+00 | 2.48E+00 | 5.40E-01 |  | - |
|  |  | Inverse variance weighted | 4 | 1.55E+00 | 2.37E+00 | 5.13E-01 | 0.4417 | - |
|  |  | Simple mode | 4 | -1.75E+00 | 4.90E+00 | 7.45E-01 |  | - |
|  |  | Weighted mode | 4 | 1.64E+00 | 2.60E+00 | 5.71E-01 | - | - |
| Parental extreme longevity (95 years and older) |  | Wald ratio | 1 | 1.91E+00 | 2.43E+01 | 9.37E-01 | - | - |
| Parental longevity (mother's age at death) |  | Wald ratio | 1 | -3.28E+00 | 4.27E+00 | 4.42E-01 | - | - |
| Parental longevity (combined parental attained age, Martingale residuals) |  | MR Egger | 10 | 1.86E+00 | 3.28E+00 | 5.87E-01 | 0.9268 | 0.9621 |
|  |  | Weighted median | 10 | 1.89E+00 | 2.05E+00 | 3.58E-01 | - | - |
|  |  | Inverse variance weighted | 10 | 1.72E+00 | 1.62E+00 | 2.88E-01 | 0.9594 | - |
|  |  | Simple mode | 10 | 1.50E+00 | 3.18E+00 | 6.48E-01 | - | - |
|  |  | Weighted mode | 10 | 2.02E+00 | 2.28E+00 | 3.99E-01 | - | - |
| Parental longevity (both parents in top 10%) |  | MR Egger | 6 | 8.57E-01 | 6.14E+00 | 8.96E-01 | 0.2886 | 0.6951 |
|  |  | Weighted median | 6 | -2.57E+00 | 3.17E+00 | 4.18E-01 | - | - |
|  |  | Inverse variance weighted | 6 | -1.39E+00 | 2.77E+00 | 6.15E-01 | 0.3910 | - |
|  |  | Simple mode | 6 | -2.67E+00 | 5.09E+00 | 6.23E-01 | - | - |
|  |  | Weighted mode | 6 | -2.96E+00 | 3.53E+00 | 4.41E-01 | - | - |
| Parental longevity (father's age at death) |  | MR Egger | 5 | -6.49E+00 | 1.27E+01 | 6.46E-01 | 0.6232 | 0.7700 |
|  |  | Weighted median | 5 | -4.41E+00 | 3.18E+00 | 1.66E-01 | - | - |
|  |  | Inverse variance weighted | 5 | -2.50E+00 | 2.63E+00 | 3.42E-01 | 0.7606 | - |
|  |  | Simple mode | 5 | -4.85E+00 | 4.57E+00 | 3.49E-01 | - | - |
|  |  | Weighted mode | 5 | -4.82E+00 | 4.12E+00 | 3.07E-01 | - | - |
| Parental longevity (father's attained age) |  | MR Egger | 13 | -4.53E-01 | 4.93E+00 | 9.28E-01 | 0.5775 | 0.6047 |
|  |  | Weighted median | 13 | 3.40E+00 | 2.45E+00 | 1.66E-01 | - | - |
|  |  | Inverse variance weighted | 13 | 1.99E+00 | 1.79E+00 | 2.66E-01 | 0.6365 | - |
|  |  | Simple mode | 13 | -1.50E+00 | 4.04E+00 | 7.17E-01 | - | - |
|  |  | Weighted mode | 13 | 2.93E+00 | 3.03E+00 | 3.53E-01 | - | - |
| Parental longevity (combined parental age at death) |  | MR Egger | 6 | 5.73E+00 | 6.03E+00 | 3.96E-01 | 0.2630 | 0.2759 |
|  |  | Weighted median | 6 | -3.17E+00 | 2.30E+00 | 1.68E-01 | - | - |
|  |  | Inverse variance weighted | 6 | -1.39E+00 | 2.25E+00 | 5.37E-01 | 0.1972 | - |
|  |  | Simple mode | 6 | -4.08E+00 | 3.03E+00 | 2.35E-01 | - | - |
|  |  | Weighted mode | 6 | -3.64E+00 | 2.73E+00 | 2.40E-01 | - | - |
| Longevity | *G_Sporobacter_HB* | MR Egger | 42 | 8.37E-01 | 1.51E-01 | 2.05E-06 | 0.9850 | 0.3225 |
|  |  | Weighted median | 42 | 6.56E-01 | 9.58E-02 | 7.24E-12 | - | - |
|  |  | Inverse variance weighted | 42 | 7.02E-01 | 6.78E-02 | 4.21E-25 | 0.9835 | - |
|  |  | Simple mode | 42 | 6.11E-01 | 1.65E-01 | 6.28E-04 | - | - |
|  |  | Weighted mode | 42 | 6.79E-01 | 1.40E-01 | 1.79E-05 | - | - |
| Healthspan |  | MR Egger | 331 | -5.24E+00 | 7.01E-01 | 7.39E-13 | 1.0000 | 0.0000 |
|  |  | Weighted median | 331 | -1.03E+00 | 2.52E-01 | 4.61E-05 | - | - |
|  |  | Inverse variance weighted | 331 | -8.70E-01 | 1.71E-01 | 3.62E-07 | 0.9870 | - |
|  |  | Simple mode | 331 | -9.15E-01 | 8.55E-01 | 2.85E-01 | - | - |
|  |  | Weighted mode | 331 | -9.99E-01 | 6.00E-01 | 9.71E-02 | - | - |
| Lifespan |  | MR Egger | 431 | 3.90E+00 | 4.23E-01 | 1.54E-18 | 0.6013 | 0.0000 |
|  |  | Weighted median | 431 | 1.73E+00 | 2.59E-01 | 2.35E-11 | - | - |
|  |  | Inverse variance weighted | 431 | 1.31E+00 | 1.63E-01 | 9.53E-16 | 0.1236 | - |
|  |  | Simple mode | 431 | 1.87E+00 | 1.05E+00 | 7.40E-02 | - | - |
|  |  | Weighted mode | 431 | 2.14E+00 | 6.93E-01 | 2.15E-03 | - | - |
| Parental longevity (combined parental age at death) |  | MR Egger | 6 | 1.51E+01 | 5.08E+00 | 4.13E-02 | 0.5394 | 0.0593 |
|  |  | Weighted median | 6 | 1.68E+00 | 2.84E+00 | 5.55E-01 | - | - |
|  |  | Inverse variance weighted | 6 | 2.63E+00 | 2.50E+00 | 2.92E-01 | 0.0772 | - |
|  |  | Simple mode | 6 | -9.96E-01 | 5.39E+00 | 8.61E-01 | - | - |
|  |  | Weighted mode | 6 | -1.78E+00 | 5.10E+00 | 7.41E-01 | - | - |
| Parental longevity (combined parental attained age, Martingale residuals) |  | MR Egger | 10 | -7.63E+00 | 3.11E+00 | 3.96E-02 | 0.8539 | 0.0622 |
|  |  | Weighted median | 10 | -9.98E-01 | 2.18E+00 | 6.46E-01 | - | - |
|  |  | Inverse variance weighted | 10 | -1.78E+00 | 1.54E+00 | 2.47E-01 | 0.4629 | - |
|  |  | Simple mode | 10 | 9.79E-01 | 4.24E+00 | 8.22E-01 | - | - |
|  |  | Weighted mode | 10 | -6.41E+00 | 2.49E+00 | 3.01E-02 | - | - |
| Parental longevity (both parents in top 10%) |  | MR Egger | 6 | 1.42E+01 | 5.22E+00 | 5.26E-02 | 0.7473 | 0.0908 |
|  |  | Weighted median | 6 | 5.05E+00 | 3.35E+00 | 1.32E-01 | - | - |
|  |  | Inverse variance weighted | 6 | 4.17E+00 | 3.02E+00 | 1.68E-01 | 0.2314 | - |
|  |  | Simple mode | 6 | 2.04E+00 | 7.42E+00 | 7.94E-01 | - | - |
|  |  | Weighted mode | 6 | 8.54E+00 | 3.30E+00 | 4.91E-02 | - | - |
| Parental longevity (mother's attained age) |  | MR Egger | 4 | -1.75E+01 | 9.91E+00 | 2.19E-01 | 0.1215 | 0.2758 |
|  |  | Weighted median | 4 | -4.59E+00 | 2.45E+00 | 6.13E-02 |  | - |
|  |  | Inverse variance weighted | 4 | -3.60E+00 | 3.84E+00 | 3.48E-01 | 0.0311 | - |
|  |  | Simple mode | 4 | 2.46E+00 | 7.86E+00 | 7.75E-01 | - | - |
|  |  | Weighted mode | 4 | -6.28E+00 | 2.54E+00 | 8.98E-02 | - | - |
| Parental extreme longevity (95 years and older) |  | Wald ratio | 1 | 5.17E+01 | 2.51E+01 | 3.90E-02 | - | - |
| Parental longevity (mother's age at death) |  | Wald ratio | 1 | 1.10E+01 | 4.04E+00 | 6.41E-03 | - | - |
| Parental longevity (father's age at death) |  | MR Egger | 5 | 2.21E+01 | 1.72E+01 | 2.90E-01 | 0.1093 | 0.3048 |
|  |  | Weighted median | 5 | -1.96E+00 | 3.51E+00 | 5.76E-01 | - | - |
|  |  | Inverse variance weighted | 5 | 1.27E+00 | 3.78E+00 | 7.38E-01 | 0.0581 | - |
|  |  | Simple mode | 5 | -3.43E+00 | 4.13E+00 | 4.53E-01 | - | - |
|  |  | Weighted mode | 5 | -3.17E+00 | 4.41E+00 | 5.12E-01 | - | - |
| Parental longevity (father's attained age) |  | MR Egger | 13 | -1.12E+01 | 6.12E+00 | 9.53E-02 | 0.0607 | 0.1413 |
|  |  | Weighted median | 13 | 7.55E-01 | 2.68E+00 | 7.78E-01 | - | - |
|  |  | Inverse variance weighted | 13 | -2.14E+00 | 2.38E+00 | 3.68E-01 | 0.0248 | - |
|  |  | Simple mode | 13 | 2.47E+00 | 4.97E+00 | 6.27E-01 | - | - |
|  |  | Weighted mode | 13 | 2.01E+00 | 4.26E+00 | 6.45E-01 | - | - |
| Longevity | *G_Veillonella_HB* | MR Egger | 42 | -3.23E-02 | 1.10E-01 | 7.71E-01 | 1.0000 | 0.0278 |
|  |  | Weighted median | 42 | 1.33E-01 | 6.55E-02 | 4.19E-02 | - | - |
|  |  | Inverse variance weighted | 42 | 1.92E-01 | 4.97E-02 | 1.11E-04 | 0.9999 | - |
|  |  | Simple mode | 42 | 8.32E-02 | 1.08E-01 | 4.46E-01 | - | - |
|  |  | Weighted mode | 42 | 1.01E-01 | 8.86E-02 | 2.60E-01 | - | - |
| Healthspan |  | MR Egger | 331 | -5.17E-01 | 9.16E-01 | 5.73E-01 | 0.0000 | 0.0448 |
|  |  | Weighted median | 331 | 3.56E-01 | 2.07E-01 | 8.50E-02 | - | - |
|  |  | Inverse variance weighted | 331 | 1.27E+00 | 2.22E-01 | 9.69E-09 | 0.0000 | - |
|  |  | Simple mode | 331 | 8.48E-01 | 4.92E-01 | 8.55E-02 | - | - |
|  |  | Weighted mode | 331 | 2.68E-01 | 3.77E-01 | 4.78E-01 | - | - |
| Lifespan |  | MR Egger | 431 | -7.52E-01 | 3.11E-01 | 1.61E-02 | 1.0000 | 0.0001 |
|  |  | Weighted median | 431 | 1.78E-01 | 1.71E-01 | 2.97E-01 | - | - |
|  |  | Inverse variance weighted | 431 | 4.29E-01 | 1.15E-01 | 1.83E-04 | 1.0000 | - |
|  |  | Simple mode | 431 | -4.46E-01 | 5.04E-01 | 3.76E-01 | - | - |
|  |  | Weighted mode | 431 | 2.79E-02 | 3.48E-01 | 9.36E-01 | - | - |
| Parental longevity (mother's attained age) |  | MR Egger | 4 | -1.30E+00 | 9.43E+00 | 9.03E-01 | 0.0286 | 0.9848 |
|  |  | Weighted median | 4 | -7.35E-01 | 1.75E+00 | 6.74E-01 | - | - |
|  |  | Inverse variance weighted | 4 | -1.50E+00 | 2.52E+00 | 5.53E-01 | 0.0685 | - |
|  |  | Simple mode | 4 | -4.56E-01 | 2.53E+00 | 8.69E-01 | - | - |
|  |  | Weighted mode | 4 | -6.56E-01 | 1.86E+00 | 7.47E-01 | - | - |
| Parental extreme longevity (95 years and older) |  | Wald ratio | 1 | -3.20E+00 | 1.77E+01 | 8.57E-01 | - | - |
| Parental longevity (mother's age at death) |  | Wald ratio | 1 | 1.10E+00 | 2.97E+00 | 7.12E-01 | - | - |
| Parental longevity (combined parental attained age, Martingale residuals) |  | MR Egger | 10 | 1.00E+00 | 2.29E+00 | 6.72E-01 | 0.9398 | 0.4652 |
|  |  | Weighted median | 10 | -6.21E-01 | 1.44E+00 | 6.67E-01 | - | - |
|  |  | Inverse variance weighted | 10 | -5.24E-01 | 1.13E+00 | 6.42E-01 | 0.9412 | - |
|  |  | Simple mode | 10 | -7.19E-01 | 2.22E+00 | 7.54E-01 | - | - |
|  |  | Weighted mode | 10 | -4.31E-01 | 1.67E+00 | 8.02E-01 | - | - |
| Parental longevity (both parents in top 10%) |  | MR Egger | 6 | -2.63E-01 | 3.83E+00 | 9.48E-01 | 0.5876 | 0.7577 |
|  |  | Weighted median | 6 | 4.36E-01 | 2.23E+00 | 8.45E-01 | - | - |
|  |  | Inverse variance weighted | 6 | 8.37E-01 | 1.89E+00 | 6.58E-01 | 0.7102 | - |
|  |  | Simple mode | 6 | -1.57E+00 | 3.64E+00 | 6.83E-01 | - | - |
|  |  | Weighted mode | 6 | 6.05E-01 | 2.30E+00 | 8.03E-01 | - | - |
| Parental longevity (father's age at death) |  | MR Egger | 5 | -4.24E+00 | 8.87E+00 | 6.65E-01 | 0.4261 | 0.6318 |
|  |  | Weighted median | 5 | -1.14E+00 | 2.26E+00 | 6.15E-01 | - | - |
|  |  | Inverse variance weighted | 5 | 3.70E-01 | 1.83E+00 | 8.40E-01 | 0.5467 | - |
|  |  | Simple mode | 5 | -1.24E+00 | 3.09E+00 | 7.08E-01 | - | - |
|  |  | Weighted mode | 5 | -1.07E+00 | 2.73E+00 | 7.14E-01 | - | - |
| Parental longevity (father's attained age) |  | MR Egger | 13 | 3.90E+00 | 3.44E+00 | 2.81E-01 | 0.5131 | 0.3545 |
|  |  | Weighted median | 13 | 1.99E+00 | 1.75E+00 | 2.56E-01 | - | - |
|  |  | Inverse variance weighted | 13 | 8.01E-01 | 1.25E+00 | 5.22E-01 | 0.5180 | - |
|  |  | Simple mode | 13 | 2.88E+00 | 2.63E+00 | 2.95E-01 | - | - |
|  |  | Weighted mode | 13 | 1.98E+00 | 2.20E+00 | 3.87E-01 | - | - |
| Parental longevity (combined parental age at death) |  | MR Egger | 6 | 1.00E+00 | 3.69E+00 | 7.99E-01 | 0.4779 | 0.8382 |
|  |  | Weighted median | 6 | -6.61E-03 | 1.66E+00 | 9.97E-01 | - | - |
|  |  | Inverse variance weighted | 6 | 2.49E-01 | 1.29E+00 | 8.47E-01 | 0.6163 | - |
|  |  | Simple mode | 6 | -5.01E-01 | 2.45E+00 | 8.46E-01 | - | - |
|  |  | Weighted mode | 6 | -9.87E-03 | 2.11E+00 | 9.96E-01 | - | - |

***Note*:** G, genus; RNT, rank-normal transformation; HB, hurdle binary; b means the estimated causal effect.
